# Shared effects of one’s own and others’ experiences during reinforcement learning on episodic memory

**DOI:** 10.1038/s41539-026-00409-7

**Published:** 2026-02-28

**Authors:** Maria A. Woitow, Anthony I. Jang, Ben Eppinger, Matthew R. Nassar, Marcel Brass, Julia M. Rodriguez Buritica

**Affiliations:** 1 Berlin School of Mind and Brain & Department of Psychology, Humboldt-Universität zu Berlin, Berlin, Germany; 2 Department of Psychiatry and Biobehavioral Sciences, Jane and Terry Semel Institute for Neuroscience and Human Behavior, University of California Los Angeles, Los Angeles, CA USA; 3 Department of Psychology, University of Greifswald, Greifswald, Germany; 4 Department of Psychology, Concordia University, Montreal, QC Canada; 5 Robert J. and Nancy D. Carney Institute for Brain Science, Brown University, Providence, RI USA; 6 Department of Neuroscience, Brown University, Providence, RI USA; 7 Science of Intelligence, Research Cluster of Excellence, Berlin, Germany; 8 Department of Experimental Psychology, Ghent University, Ghent, Belgium

**Keywords:** Human behaviour, Learning and memory, Reward

## Abstract

Humans learn not only from their own experiences but also by observing others. Prior research has shown that reward prediction errors (RPEs) – the difference between expected and received outcomes – guide both experiential and observational reinforcement learning. While RPEs from direct experience have been linked to memory formation, it remains unclear whether vicarious RPEs play a similar role in observational learning. Using an incidental memory paradigm, we investigated how experiential and observational learning in a decision-making task shape memory and examined the role of RPEs in this process. Although recognition accuracy did not differ between learning conditions, participants reported higher confidence in memories from experiential trials. Notably, across both learning conditions, gambling and positive RPEs during memory item presentation were associated with enhanced memory. These findings advance our understanding of how observing others’ choices and outcomes affects episodic memory by emphasizing shared encoding mechanisms with experiential learning.

## Introduction

Across diverse learning domains (e.g., motor skill learning, knowledge acquisition, or socio-emotional learning) and in informed decision-making, individuals benefit not only from their own experiences but also from observing others’ behavior. One important advantage of observational learning is that it allows individuals to learn without direct interaction with the environment, reducing effort and avoiding the potential risks associated with trial-and-error learning. However, this may also come with a disadvantage, because the memory representations formed during observational learning are often less rich and vivid than those generated through direct, interactive experience^1^. Consequently, the common view in the psychology literature is that active (i.e., experiential) learning, as compared to passive (i.e., observational) learning, leads to better memory encoding in many contexts^1–4^.

Recent neurocomputational studies in decision-making and reinforcement learning have shown comparable learning effects (e.g., learning action-outcome associations) following experiential and observational conditions^5–7^ and proposed shared underlying processes^8–11^. Other studies have suggested that memory differences between experiential and observational learning depend on both the context and the type of memory tested. For example, when comparing active drivers and passive passengers in a virtual reality task, Plancher et al.^12^ found better spatial memory in the active condition but better incidental memory for objects encountered on the route in the observational condition. In addition, a review by Steffens et al.^13^ concluded that whether enactment and pure observation differ in subsequent memory performance depends on the experimental design and the type of memory tested (i.e., cued recall versus free recall).

To better understand the processes involved in reinforcement learning and how these relate to later memory, previous research has employed neurocomputational approaches. A crucial computational learning signal during reinforcement learning is the reward prediction error (RPE), which reflects the difference between the expected and the received reward^14–16^. RPEs signal whether stored information about the value of actions and experiences needs to be updated. This signal is thought to be coded via midbrain dopamine neurons^15,17–19^, where dopamine release increases when the outcome exceeds expectations and decreases when the outcome is worse than expected, reflecting a signed RPE^20–23^. Over the course of learning, this dopaminergic signal becomes associated with the earliest reward-predictive cue^15^. Several studies investigated the relationship between RPEs and memory encoding^24–29^. While some evidence suggests a negative association between RPEs and memory^30^, most studies revealed that larger RPEs are linked to enhanced memory performance^24,27,29,31,32^. A recent study by Jang et al. ^26^ highlighted the computationally and temporally precise role of RPEs in enhancing memory encoding. The authors found that memory formation was proportional to RPEs elicited during the presentation of the memory item. While several studies examined the effect of RPE signaling during experiential learning in memory formation, to our knowledge, no study has yet investigated this link for vicarious RPEs generated during observational learning. Hence, it remains unclear whether the effects of observational RPEs on memory performance are comparable to experienced RPEs. Given previous research suggested that both experiential and observational reinforcement learning are driven by RPEs^8,10,11^, investigating the relationship between vicarious RPEs and memory would help to better understand how these learning processes shape memory.

**Fig. 1:**
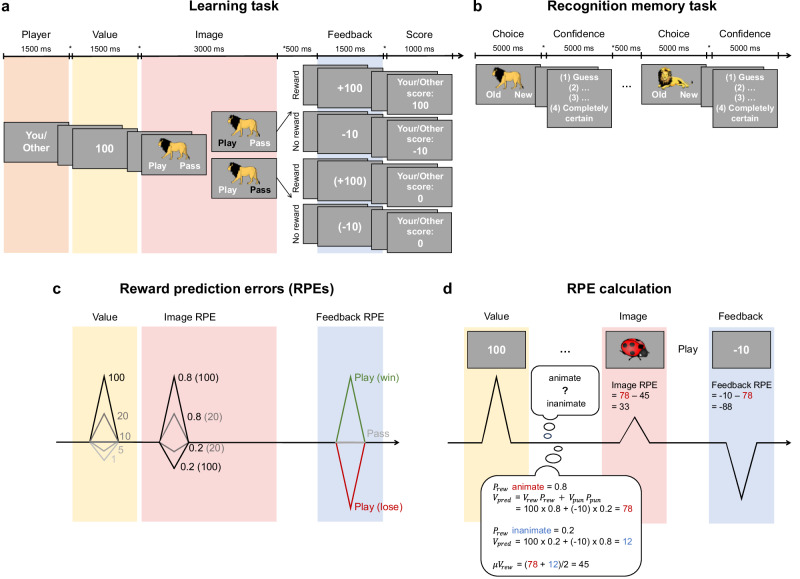
Task procedure and reward predictions. **a** Learning task. At the start of each trial, the learning condition was cued: “You” – experiential learning (EL), “Other” – observational learning (OL), followed by the trial value (potential reward; e.g., 100). Next, a unique image, reflecting the reward probability, was shown, and participants chose to play or to pass (EL) or observed a play-or-pass choice by the other player (OL). Then, participants (OL: other player) saw their earnings when playing (win or lose points) or the hypothetical outcome when passing. At the end of each trial, the current player’s cumulative score is shown. **b** Recognition memory task. In a surprise memory test, participants saw the images from the learning task mixed with foils and judged whether they were old or new before rating their confidence. **c** Reward prediction errors (RPEs). The trial value is equally sampled from [1, 5, 10, 20, 100]. The image RPE results from the reward likelihood (e.g., 0.8 and 0.2) and magnitude (e.g., 100 and 20). The feedback RPE depends on the trial outcome (win or lose). **d** Example for RPE calculation. The trial value indicates whether the reward magnitude is better or worse than the expected average (10). The image RPE is the difference between the expected reward of the shown image category (e.g., 78) and the reward prediction before seeing the image (average across categories, e.g., 45). The predicted reward per image category is computed from the value and probability of reward and punishment. The feedback RPE is the difference between the received and expected outcome.

To address this question, the current study investigates how experiential and observational learning influence episodic memory by comparing their underlying computational processes. We employed an incidental memory paradigm^26^ consisting of a learning and decision-making task involving interleaved experiential and observational trials, followed by an unexpected memory test (Fig. 1a, b).

A total of 79 young adults (52 females, 18 to 30 years old) completed the experiment. In the learning task, participants learned reward probabilities associated with image categories through both their own (experiential learning, EL) and another player’s (observational learning, OL; computer generated) choices (Fig. 1a). On each trial, participants either accepted (“Play”) or rejected (“Pass”) the opportunity to gamble (EL) or observed such a choice by another player (OL). Their goal was to optimize choice behavior to maximize monetary payout at the end of the experiment. The learning condition was indicated at the beginning of each trial (Fig. 1a, orange shading; “You” – EL, “Other” – OL), followed by a value representing the magnitude of the potential reward (Fig. 1a, yellow shading). The probability of receiving this reward was then signaled by a trial-unique image (Fig. 1a, red shading) belonging to one of two possible categories (animate or inanimate). These two categories were associated with inversely linked reward probabilities that changed repeatedly during the task while maintaining their inverse relationship. Because participants were given no explicit information about the reward probabilities, they had to learn them from trial-to-trial feedback. During the image presentation, participants (other player in OL trials) made a play-or-pass choice. Reward delivery was determined by the probability associated with the presented image and was revealed in the subsequent feedback phase (Fig. 1a, blue shading). Informative feedback was provided on each trial, regardless of the play-or-pass choice, to enable reward probability learning.

Following the learning task, participants were tested on their memory for the images presented in both learning conditions (Fig. 1b). During this test, they viewed all images from the learning task intermixed with an equal number of semantically matching foils. For each image, participants indicated whether it was old (seen before) or new (not seen before) and then rated their confidence on a scale of 1 to 4.

Reward prediction errors (RPEs) were computed at three key stages—reward magnitude presentation, image presentation, and feedback—and analyzed to assess how fluctuations in RPEs impact subsequent memory performance. The first time point was the presentation of the potential reward magnitude (trial value; Fig. 1c, yellow shading). Values larger than the average trial value reflected a positive RPE, whereas smaller values reflected a negative RPE. The second RPE was elicited by the image (image RPE) and indicated whether the probability of reward delivery was better or worse than the average reward probability across both image categories (Fig. 1c, red shading). An image from the more frequently rewarded category would elicit a positive RPE, while an image from the less frequently rewarded category would yield a negative RPE. The final RPE occurred during feedback presentation (feedback PE), reflecting whether a reward was delivered (winning or losing points) and its magnitude (Fig. 1c, blue shading).

In line with Jang et al.^26^, we expect better memory for trials in the experiential learning condition where participants decided to gamble for potential rewards. Regarding computational processes, we predict a memory-enhancing effect of positive RPEs elicited during memory item presentation when gambling in experiential trials. We assume similar computational processes are involved in experiential and observational reinforcement learning. However, due to the lack of literature, it is not clear whether we will find a similar effect of observed gambling and vicarious RPEs on memory, and hence, this will be explored.

## Results

### Gambling behavior and reinforcement learning modeling

**Fig. 2:**
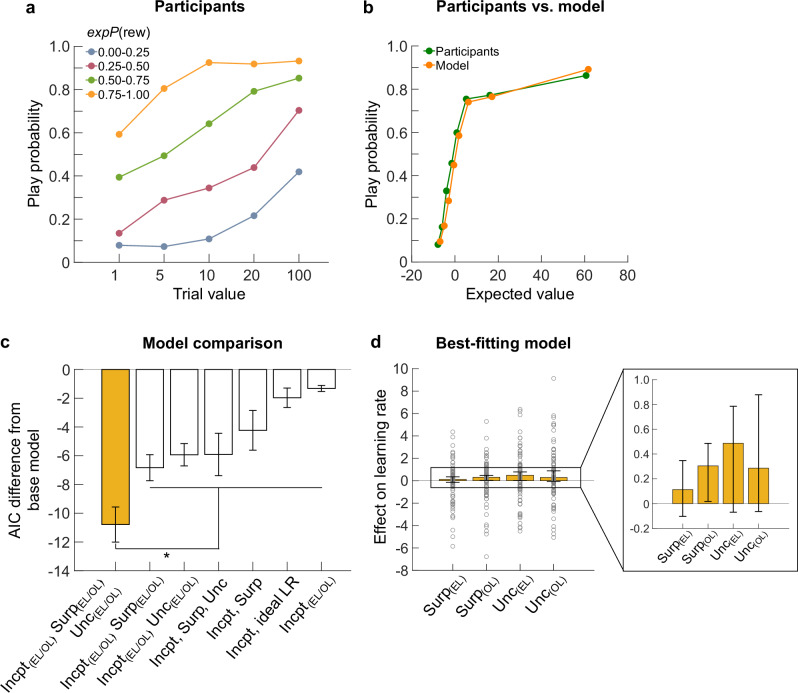
Participants’ gambling behavior and reinforcement learning (RL) model comparison. **a** Participants’ (*n* = 79) gambling behavior in experiential learning (EL) trials. The probability of playing is plotted over trial values [1, 5, 10, 20, 100] and expected reward probability (expP(rew)). **b**–**d**. RL modeling. **b** Participants’ play probability (EL) vs. RL model-derived play probability for the same trials (EL). **c** Comparison of eight different RL models using the Akaike Information Criterion (AIC). Bars show the mean difference in AIC between each model and the base model, which includes only the intercept term for the learning rate parameter. Each model has different parameters that affect the learning rate (Intcpt = learning rate intercept term, Surp = surprise, Unc = uncertainty, Ideal LR = learning rate computed from the ideal observer model). Parameters labeled EL/OL denote those fit separately for the EL and OL trials, respectively. The best-fitting model (yellow) had a significantly lower AIC when compared to all other models (*p < 0.001 across all pairwise comparisons between the yellow bar and other bars). **d** Median maximum likelihood estimates of surprise and uncertainty parameters of the best-fitting model.

The analysis of participants’ gambling behavior revealed that the likelihood of choosing to play increased as a function of both trial value and expected reward probability (Fig. 2a). Participants’ decisions to gamble depended on the reward magnitude (*trial value*: *ß* = 1.35, *z* = 8.02, *p* < 0.001), reward probability (*expP(rew)*: *ß* = 1.42, *z* = 33.49, *p* < 0.001), and their interaction (*trial value x expP(rew): ß* = 0.24, *z* = 4.37, *p* < 0.001; Supplementary Table 1). These findings indicate that participants attended closely to trial information and adjusted their behavior accordingly.

To analyze trial-to-trial dynamics in gambling behavior, we fitted several reinforcement learning (RL) models directly to participants’ choices. The model that best accounted for participants’ behavior (Fig. 2b, c) separated the learning rate for experiential (EL) and observational learning (OL) trials, suggesting that surprise and uncertainty influenced the learning rate differently across learning conditions. Overall, the AIC was significantly lower for this model compared to all other models (*p* < 0.001, across all pairwise comparisons).

To explore this further, we examined the median maximum likelihood estimates for surprise and uncertainty in the best-fitting model (Fig. 2d; for parameter recovery, see Supplementary Figs. 1,2). While all four parameters trended above zero, none reached statistical significance (Surp EL: median = 0.11, *p* = 0.87; Surp OL: median = 0.30, *p* = 0.19; Unc EL: median = 0.49, *p* = 0.06; Unc OL: median = 0.29, *p* = 0.06; Wilcoxon signed-rank t-tests against 0). In addition, surprise and uncertainty estimates did not differ between the EL and OL conditions (Surp: *p* = 0.34, Unc: *p* = 0.97; Wilcoxon signed-rank tests). In summary, the RL modeling results indicate that participants were more responsive to feedback that was surprising or occurred during periods of uncertainty. Moreover, participants modulated their learning rates and, therefore gambling behavior across experiential and observational trials. However, the parameter estimates suggest that these modulations were not consistent across individuals.

### Memory performance

**Fig. 3:**
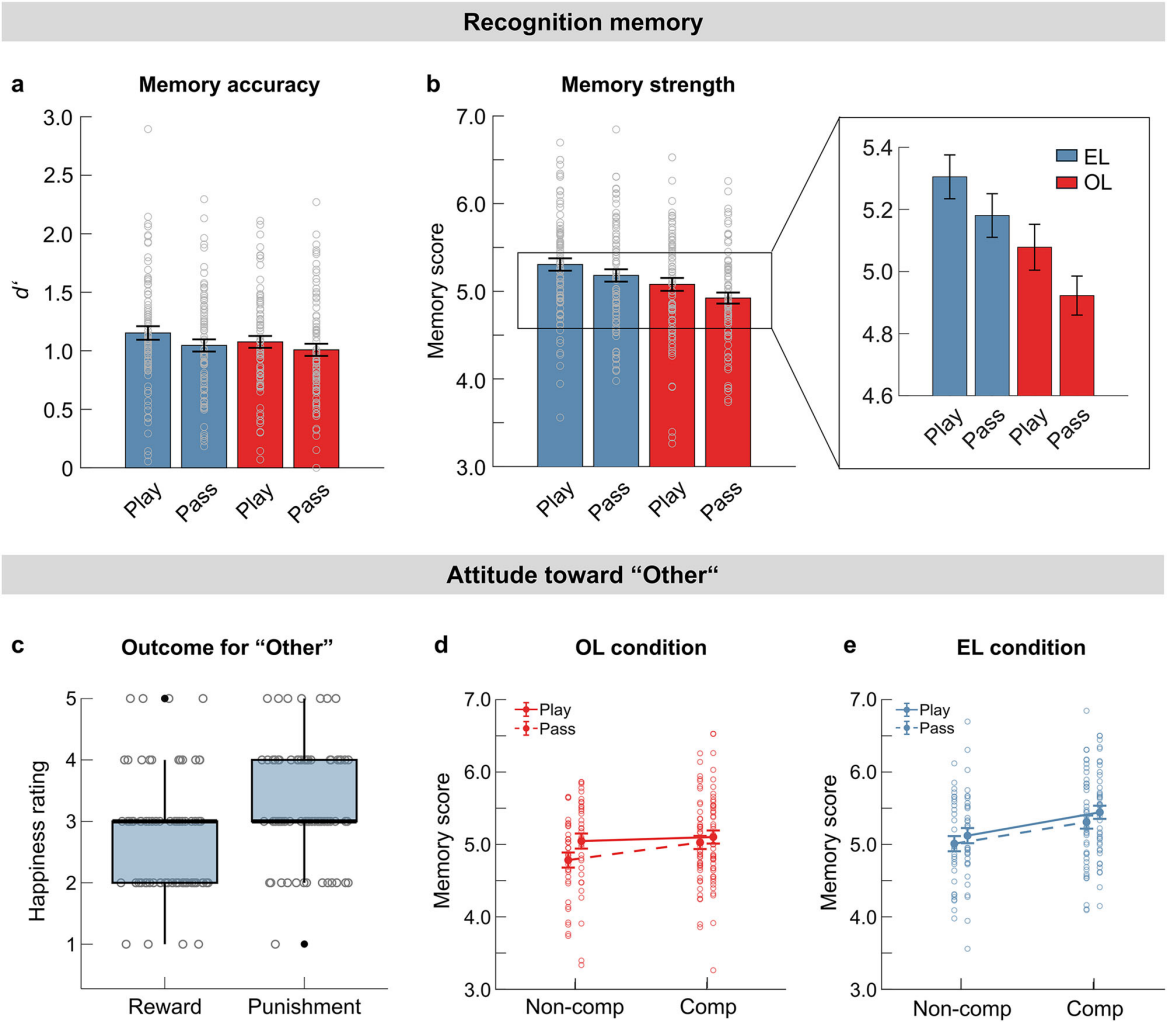
Behavioral effects. **a**–**e**. Plots contain individual data points (each circle represents one participant, n = 79). **a**, **b** Recognition memory. **a** Memory accuracy (*d’, d prime*). Average (mean ± SEM) *d’* values for play and pass trials per learning condition (EL, OL). **b** Memory strength. Average (mean ± SEM) memory score (old) for play and pass trials per learning condition (EL, OL). **c**–**e** Participants’ attitude toward the other player. **c** Participants’ happiness ratings (1 – very unhappy, 5 – very happy) from a debriefing questionnaire for a good outcome (reward) versus a bad outcome (punishment) for the other player (OL). **d** Memory scores (mean ± SEM) for competitive (Comp) and non-competitive (Non-comp) participants in OL condition split by play and pass trials. **e** Memory scores (mean ± SEM) for competitive (Comp) and non-competitive (Non-comp) participants in EL condition split by play and pass trials.

Memory performance was first assessed by analyzing the participants’ ability to distinguish between target images and matching foils. Participants’ recognition accuracy was positively influenced by the decision to gamble (Fig. 3a; *choice*: *ß* = 0.04, *t* = 2.26, *p* = 0.025). Notably, this effect did not differ between learning conditions (*learning condition x choice*: *ß* = 0.01, *t* = 0.50, *p* = 0.62). Moreover, we found no significant main effect of learning condition (*ß* = 0.03, *t* = 1.48, *p* = 0.14, Supplementary Table 2). These results suggest that participants’ ability to distinguish targets from foils did not substantially differ between experiential and observational learning and was enhanced by both experienced and observed gambling. The effect of gambling was further supported by higher hit rates in play trials (*choice: ß* = 0.02, *t* = 3.47, *p* < 0.001), whereas false-alarm rates did not differ between choice trials (*choice: ß* = 0.007, *t* = 0.20, *p* = 0.84). There was no evidence that these effects varied across learning conditions (*learning condition x choice;* hits: *ß* = −0.005, *t* = −1.04, *p* = 0.30, false alarms: *ß* = −0.004, *t* = −1.18, *p* = 0.24). The absence of a difference in overall recognition accuracy between learning conditions reflected both higher hit rates and false-alarm rates in experiential trials (*learning condition:* hits: *ß* = 0.02, *t* = 4.62, *p* < 0.001; false alarms: *ß* = 0.01, *t* = 2.76, *p* = 0.006; Supplementary Table 3,4, Supplementary Fig. 3).

When considering participants’ confidence ratings (scale of 1 to 4) for their old-or-new judgments, higher accuracy was associated with greater confidence (ratings of 3 or 4; *confidence bin: ß* = 0.38, *t* = 16.86, *p* < 0.001). This effect did not differ between learning trials (*learning condition x confidence bin*: *ß* = −0.009, *t* = −0.42, *p* = 0.68, Supplementary Table 5). These results indicate that subjective confidence ratings reflect true memory strength in both learning conditions.

In the next step, participants’ memory strength was assessed by analyzing the memory score (scale of 1 to 8) which integrates old-or-new responses with confidence ratings. Following Jang et al.^26^, we expected higher scores in play than pass trials during experiential learning. Consistent with this prediction, our analysis showed that memory strength depended on the context in which images were presented (Fig. 3b). Specifically, memory scores were higher in the EL condition (*learning condition*: *ß* = 0.12, *t* = 5.35, *p* < 0.001) and for play trials (*choice*: *ß* = 0.07, *t* = 3.10, *p* = 0.002) with no evidence for an interaction (*learning condition x choice*: *ß* = −0.008, *t* = −0.34, *p* = 0.73, Supplementary Table 6). These results show that participants remembered images from experiential trials more confidently than those from observational trials. Moreover, memory strength was higher when the gamble was accepted (play) compared to rejected (pass), independent of the learning condition.

### Debriefing questionnaire

In an explorative analysis, we investigated participants’ attitudes toward the other player observed in OL trials. Debriefing questionnaire responses indicated that, on average, participants reported being rather unhappy when the other player received a good outcome (Fig. 3c; reward; *p* = 0.011; Wilcoxon signed-rank test against neutral rating of 3) and rather happy when this player obtained a bad outcome (punishment; *p* = 0.004). Ratings for the two outcome types differed significantly from each other (*p* = 0.003; Wilcoxon signed-rank test), suggesting that the task elicited competitive feelings toward the observed player, or at least social comparison.

Participants were then categorized as competitive (rating: punishment > reward) or non-competitive (rating: punishment ≤ reward), and we tested whether this attitude influenced memory performance. In OL trials (Fig. 3d), there was no evidence for an effect on memory strength (*attitude*: *ß* = 0.08, *t* = 1.17, *p* = 0.25; α_corrected_ = 0.025; Supplementary Table 7). In contrast, in EL trials (Fig. 3e), competitiveness predicted memory performance (*attitude*: *ß* = 0.16, *t* = 2.50, *p* = 0.015, α_corrected_ = 0.025) across choice trials (*attitude x choice*: *ß* = 0.006, *t* = 0.20, *p* = 0.85; Supplementary Table 8). These results indicate that competitive feelings were associated with enhanced memory formation during experiential but not observational learning.

### Reward prediction errors and memory

The aim of the computational analysis was to explore the relationship between reinforcement learning processes and subsequent memory. Specifically, we examined the effect of reward prediction errors (RPE; the difference between the predicted and the obtained reward^14^) and related computational parameters elicited during the learning task (i.e., trial value, image RPE, feedback RPE) on memory strength using linear regression analyses. The direct influence of these parameters on memory was assessed by conducting one-sample t-tests against 0 on the slopes of their relationship.

**Fig. 4:**
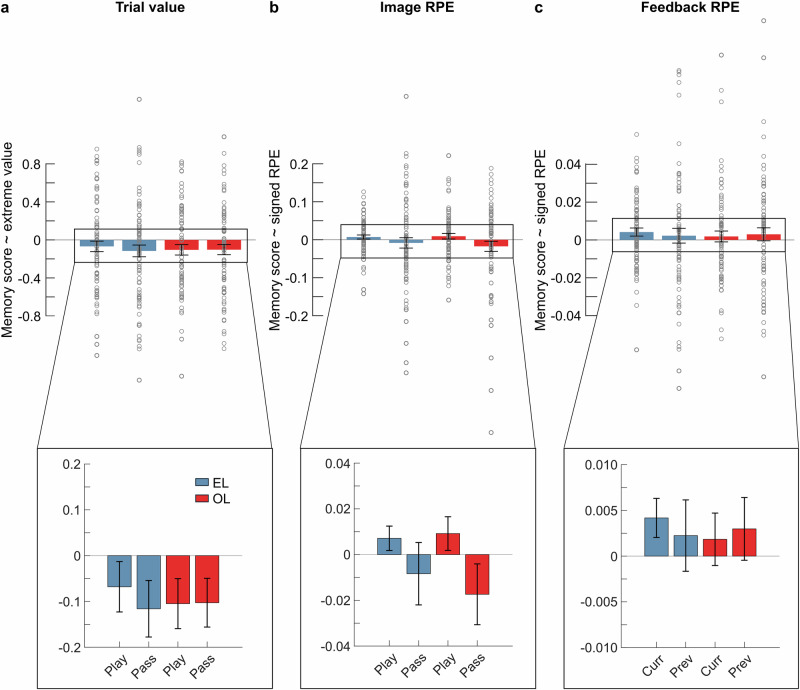
Effects of trial value and reward prediction errors (RPEs) on memory strength. **a**–**c** The plots contain individual data points (each grey circle represents one participant, n = 79). **a** Effect of extreme trial value. Trial values were binned based on their relative deviation from the average trial value 10 (bin 1:10, bin 2:5 & 20, bin 3:1 & 100). Average slopes (mean ± SEM) between memory scores and more extreme values (binned trial values) per learning condition (EL, OL) and choice (Play, Pass). **b** Effect of signed image RPE. Image RPEs were binned based on their valence (bin 1 < 0, bin 2 > 0). Average slopes (mean ± SEM) between memory scores and binned image RPEs per learning condition (EL, OL) and choice (Play, Pass). **c** Effect of signed feedback RPE. Feedback RPEs were binned based on their valence (bin 1 < 0, bin 2 > 0). Average slopes (mean ± SEM) between memory scores and binned feedback RPEs for play trials are plotted per learning condition (EL, OL) and trial of interest (Curr - current, Prev - previous). Current - effect of feedback RPE on memory for the same trial, previous - effect of feedback RPE on memory for the next trial.

First, we assessed whether trial values (i.e., magnitude of potential reward) influenced memory. We found no linear effect in either condition (*ps* ≥ 0.02; α_corrected_ = 0.0125; Supplementary Table 9). Inspection of the slopes suggests a potential inverted-U shaped relationship (Supplementary Fig. 4). Hence, we next examined the impact of more extreme trial values (i.e., values deviating from the average expected trial value of 10) on memory (Fig. 4a), which revealed no significant effect in either condition (*ps* ≥ 0.06, α_corrected_ = 0.0125; Supplementary Table 10). However, when analyzing the influence of learning condition and choice on these slopes, we observed a significant negative association between extreme trial values and memory (*ß0* = −0.1, *t* = −3.48, *p* < 0.001) across learning conditions (*learning condition*: *ß* = 0.006, *t* = 0.21, *p* = 0.83) and choice trials (*choice*: *ß* = 0.01, *t* = 0.41, *p* = 0.68; *learning condition x choic*e: *ß* = 0.01, *t* = 0.45, *p* = 0.66; Supplementary Table 11). These results indicate that memory strength was reduced for trials in which the potential reward was either higher or lower than the average trial value (10), independent of the learning condition and play-or-pass choices.

We examined the influence of image RPEs, indicating whether the reward prediction was better or worse than expected, on memory. Following Jang et al.^26^, we predicted a positive relationship in the EL condition for play but not pass trials. Although there was a numerical trend in the predicted direction, which did not differ substantially between learning conditions (Fig. 4b; Supplementary Fig. 5), we found no direct effect of signed image RPEs on memory (*ps* ≥ 0.19; α_corrected_ = 0.0125; Supplementary Table 12). Notably, gambling positively modulated the relationship between image RPEs and memory (*choice*: *ß* = 0.01, *t* = 2.02, *p* = 0.045) independent of the learning condition (*learning condition*: *ß* = 0.002, *t* = 0.33, *p* = 0.74; *learning condition x choice*: *ß* = −0.003, *t* = −0.53, *p* = 0.60; Supplementary Table 13). Moreover, participants’ sensitivity to reward magnitude and probability, which modulate the image RPE, positively correlated with the effect of this RPE on memory (Spearman’s *ρ* = 0.29, *p* = 0.011, *n* = 79).

In an explorative analysis, we examined memory performance separately for positive and negative image RPE trials. Trials eliciting positive RPEs in which participants gambled or observed gambling were associated with enhanced memory (EL: *t*_78_ = 2.66, *p* = 0.009, *d* = 0.30, 95% CI = 0.03–0.19; OL: *t*_78_ = 2.37, *p* = 0.02, *d* = 0.27, 95% CI = 0.01–0.16; one-sample t-tests against 0; α_corrected_ = 0.025), whereas pass trials were not (*ps* ≥ 0.07; α_corrected_ = 0.025; Supplementary Table 14). Memory performance for negative RPE trials did not differ from average in either learning condition (*ps* ≥ 0.32; Supplementary Table 15). These findings indicate that memory was selectively enhanced in gambling and observed gambling trials when positive image RPEs were elicited.

Finally, we examined the impact of feedback RPEs, which indicate whether the outcome was better or worse than expected, on memory during gambling. We found no effect of the current (t) or previous trial’s (t-1) feedback on memory in either condition (Fig. 4c; *ps* ≥ 0.054; α_corrected_ = 0.0125, Supplementary Table 16), with no indication of differences between learning conditions (curr: *t*_78_ = 0.62, *p* = 0.54, *d* = 0.07, 95% CI = −0.005−0.01; prev:*t*_78_ = -0.15, *p* = 0.89, *d* = −0.02, 95% CI = −0.01−0.009; paired-samples t-tests, α_corrected_ = 0.025).

Further, we found no effect of unsigned feedback RPEs (*ps* ≥ 0.24; α_corrected_ = 0.0125; Supplementary Table 17; Supplementary Fig. 6) or related parameters (i.e., surprise, uncertainty) on memory (*ps* ≥ 0.07; α_corrected_ = 0.0125; Supplementary Tables 18–20; Supplementary Fig. 7). Overall, these results suggest that RPEs elicited by the reward outcome did not modulate memory in either experiential or observational learning.

## Discussion

The current study employed an incidental memory paradigm to investigate whether one’s own experiences, as opposed to observing others’ experiences, differentially affect episodic memory in a decision-making and reward-learning context. We combined descriptive and computational modeling approaches to examine how experienced and vicarious reward prediction errors (RPEs) influence memory formation. Our findings highlight that the advantage of experiential over observational learning depends on the memory measure. We found that memory accuracy, reflecting participants’ ability to distinguish target images from matching foils, did not differ significantly between learning conditions. However, subjective memory strength, indicating how confident participants were in their old-or-new judgments, was higher following experiential learning. Interestingly, both memory accuracy and strength were positively influenced by enacted and observed gambling, suggesting that both experienced and observed risk-taking enhanced memory formation. Computational analyses revealed that, across learning conditions, more extreme potential rewards (i.e., trial value) were linked to reduced memory, while positive image RPEs were associated with enhanced memory. These results suggest a comparable role of experienced and vicarious RPE signaling in episodic memory encoding.

Analysis of recognition accuracy indicates that participants encoded incidental information (i.e., unique images) from both learning conditions. There was no significant difference between conditions, suggesting that they distinguished target stimuli from foils similarly well after experiential and observational learning. Examination of hits and false alarms showed that this lack of difference reflected both higher hit rates and higher false-alarm rates in the experiential compared to the observational learning condition. While participants more often correctly recognized images seen in experiential trials, they also more frequently misidentified the corresponding foils as “old”.

Subjective confidence ratings reveal a clear mnemonic advantage for experiential over observational learning. Participants’ confidence judgments suggested that they were more certain about correctly remembering images encountered during experiential trials. Given that confidence ratings in our study reflected true memory, this suggests that participants formed stronger memory traces of image details (e.g., color of an object) or contextual information (e.g., value presented before the image or feedback after the image) when learning from their own choices and feedback compared to those of others. This finding aligns with a large body of literature showing better memory following active (e.g., experiential) compared to passive (e.g., observational) learning across diverse domains^1–4^. Active learning is proposed to foster richer mental representations during encoding, which in turn facilitates later memory retrieval^1^. Here, “active” refers to having control over the learning process (i.e., self-directed learning), which includes even minimal interactions with the study material, such as selecting the next study item or rotating a novel three-dimensional object, as well as decision-making to guide information sampling^33^.

Consistent with this framework, prior research has shown that the mere act of choosing enhances both declarative and incidental memor^34–36^. Hence, the mnemonic benefit of experiential learning in our study may stem from the participant’s opportunity to actively choose between “Play” and “Pass”. The option to make a choice might have been perceived as a positive occurrence due to anticipation of potential rewards, generating a motivational signal that enhances memory formation^37–41^. Motivation has been shown to affect memory at multiple stages, including encoding and consolidation, via dopaminergic projections from the ventral tegmental area (VTA) throughout the mesolimbic circuit^39,42^.

Another factor that might have contributed to better subjective memory in the experiential condition is the participant’s attitude toward the other player. Responses to the debriefing questionnaire indicated that the reward-learning task evoked competitive feelings in more than half of the participants (57%), which was surprising given that outcomes in observational trials did not affect participants’ scores. We hypothesize that viewing the cumulative score for the simulated other player caused participants to compare it with their own. Importantly, this attitude influenced memory performance in the experiential but not observational learning condition. Competitive participants demonstrated increased memory strength for both play and pass trials, which may reflect an overall increase in attention and/or motivation. Previous research demonstrated that attention and memory encoding are closely related^43,44^. Because memory capacity is limited, attention plays a key role in determining which information is encoded and subsequently remembered. Thus, being attentive and motivated to outperform the other player would have benefited memory encoding^39,42,43^. It is therefore surprising that we did not find the same memory-enhancing effect of competitiveness in observational trials. As reward probabilities evolved across learning conditions, participants’ gambling performance would have also benefited from more closely attending to trial information in the observational condition. This would be expected to enhance memory in both learning conditions compared to non-competitive individuals. To better understand the role of competition and social comparison during memory encoding, future studies should include a direct measure, for example, by explicitly asking participants whether they perceived the task as competitive.

While the mnemonic benefit of one’s own experiences appears to depend on the memory measure, choosing to play increased both memory accuracy and memory strength. We observed better memory for trials where participants decided to gamble for potential rewards compared to trials where they rejected the gamble. We assume that participants anticipated the uncertain reward or punishment when gambling, whereas passing ensured that they maintained their current score. Thus, choosing to play, in contrast to passing, reflected the risky choice. The beneficial effect of perceived risk on memory replicates findings by Jang et al.^26^ and aligns with other studies showing better memory encoding in contexts of uncertain feedback^32,45^. A previous fMRI study highlighted the positive effect of choice on declarative memory by demonstrating a relationship between neural activity related to choice processes (i.e., striatal activation) and memory enhancement^36^. The authors concluded that the perceived agency over the learning process, simply by making a choice, contributes to the mnemonic benefits of active learning. This positive effect of choice was replicated and extended to incidental memory in later studies^34,35^. In addition, participants may have assigned greater value, and therefore more attention, to trials where they gambled, which would have facilitated memory encoding.

Interestingly, we found no differences between learning conditions in the effect of gambling on memory performance. Similar to the experiential condition, participants remembered images from trials in which they observed a play choice better than those in which they observed a pass choice. This finding could be explained by participants representing the observed choices similarly to their own. As reward probabilities changed across the learning task and learning conditions alternated after one or two trials, participants likely benefited from closely attending to trial information from both learning conditions to facilitate learning. Consequently, they may have encoded observed and experienced choices in a similar way. This interpretation aligns with previous research showing similarities in the neural activation during the generation of one’s own action and the imagination or observation of others’ actions^46^.

Participants’ choices in the learning task indicated they closely attended to trial information and used it to optimize their gambling behavior. We further showed that the simulated behavior in observational trials aligned with participants’ choices, thus making behavior from the two learning conditions comparable. To analyze trial-to-trial dynamics in participants’ behavior, we fitted several reinforcement learning (RL) models directly to their choices. The model that best explained behavior included both surprise and uncertainty parameters, which replicates the modeling results by Jang et al.^26^ and suggests that participants’ learning rate was affected by surprise and uncertainty estimates in line with previous studies^47–49^. This indicates that participants were more responsive to surprising outcomes and feedback in periods of uncertainty.

Moreover, we examined whether participants updated their learning rate differently depending on the learning condition. To test this, we fitted modified RL models that allowed the effects of surprise and/or uncertainty on the learning rate to vary by condition. Modeling both surprise and uncertainty separately for each learning condition significantly improved model fits compared to all alternative models. This finding is consistent with the idea that information that was surprising and obtained under uncertainty affected learning strategies differently across conditions. However, because no significant differences were observed between learning conditions in the maximum likelihood estimates of surprise and uncertainty, we cannot make any generalizable conclusion about the differential effects of surprise and uncertainty on experiential versus observational reward-learning.

To better understand how experiential versus observational learning shapes memory, we examined how reward prediction errors (RPEs) and related parameters at different stages of each learning trial relate to subsequent memory performance.

The first reward-related cue, the trial value, indicated the magnitude of potential reward. In line with Jang et al.^26^ we found no meaningful linear effect of trial values on memory strength. However, more extreme trial values were associated with reduced memory strength across learning conditions and choice trials. Specifically, values smaller or larger than the average expected value of 10 (particularly 1 and 100) were linked to weaker memory. This negative association could be explained by participants deciding to play or to pass already upon the trial value presentation. For example, seeing the high value of 100 may have been sufficient to elicit a decision to gamble, as the potential reward outweighed the risk of losing 10 points. Consequently, the subsequent image, reflecting the likelihood of receiving this reward, may have been less relevant to the decision process and therefore less attended, leading to weaker memory for that image. Conversely, when seeing the low value of 1, participants may have decided to pass, as the potential punishment (−10) outweighed the small reward. Supporting this interpretation, participants chose to play in roughly three-quarters of the trials with a value of 100, but only in about a quarter of the trials with a value of 1. In contrast, for trials with the average value of 10, where reward and punishment magnitudes were equal, play/pass choices were approximately fifty-fifty. In these trials, participants needed to attend to the image and compare it to the running estimate of which category is currently more frequently rewarding, leading to better memory for those images compared to trials with extreme values.

Interestingly, we found no play-pass difference in the effect of extreme trial values on memory, although one might expect a differential effect in trials where participants saw the value 100 and chose to pass, and similarly in trials where participants saw the value 1 and chose to gamble. In such cases, the decision to play or pass would presumably depend on the current reward probability, leading to increased attention to images from those trials. The lack of play-pass effect may be explained by the substantial imbalance in the number of play and pass choices for trials with values of 1 or 100 (i.e., participants rarely chose to gamble for a value of 1 and to pass for a value of 100). Future studies could consider using more continuous measures of trial values to address this discrepancy.

It is surprising that we show a comparable negative effect of extreme trial values in observational trials. Since participants did not make choices in these trials, the observed trial value should have been irrelevant to them. This effect might be explained by participants mentally representing observed and experienced information in similar ways. Because the binary choices in observational trials were derived from RL model simulations based on real human behavior, the simulated player also tended to play after a value of 100 and to pass after a value of 1. Thus, participants may have anticipated a play choice after seeing the value 100 and a pass choice after the value 1, leading to reduced attention to the subsequent image. This effect of anticipation aligns with a core assumption of the simulation hypothesis^50^, which proposes that the simulation of actions can elicit perceptual activity that resembles the activity that would have occurred during actual action performance. According to this hypothesis, thinking consists of simulated interactions with the environment based on the simulation of behavior (i.e., actions), perception, and anticipation.

The image RPE reflected whether the reward prediction was better or worse than expected. We found no direct impact of image RPEs on memory in either condition. In experiential play trials, this finding contradicts our expectation of a memory-enhancing effect, as shown by Jang et al.^26^. In experiential pass trials, however, the lack of an effect replicates their results. Consistent with Jang and colleagues’ work, we found evidence that gambling and sensitivity to trial information (i.e., trial value and reward probability, which modulate image RPEs) positively influenced the relationship between image RPEs and memory. In an exploratory analysis, we could demonstrate better memory performance in play trials when yielding positive, but no negative, image RPEs. Enhanced memory for gambling, particularly when associated with positive RPEs, is consistent with the idea that participants’ behavior reflects the influence of reward-predictive PEs on memory^26^. Our findings, therefore, add to a growing body of evidence highlighting the positive role of signed RPEs in memory formation^24–28,31^. Nonetheless, future studies should aim to clarify whether the absence of a direct effect of image RPEs in our study reflects differences in task design compared to Jang et al.^26^, whose study was conducted online with a larger sample size and included only experiential learning trials, or yet unknown factors.

Interestingly, we found no differences between learning conditions regarding the effect of image RPE on memory. Instead, both conditions showed a similar pattern of enhanced memory for play trials yielding positive RPEs. This suggests that experienced and vicarious image RPEs influence memory encoding in similar ways. Our results therefore support prior research pointing to similarities in RPE signaling during learning from one’s own and others’ choices and feedback^8–11^.

Finally, we examined the feedback RPE, which reflected whether the trial outcome was better or worse than expected. We found no evidence that preceding or subsequent feedback, or related computational parameters, influenced memory strength. These results align with prior findings regarding experiential learning^26^ and extend them by showing the same lack of an effect in observational trials. Because prior research has shown that unsigned (absolute magnitude) RPEs elicited during outcome presentation drive memory formation^29,32^, we also assessed the effect of unsigned feedback RPEs on memory. In contrast to these studies, we found no evidence for an effect in experiential (respectively observational) trials. A reason for this might be the timing of memory item presentation. Specifically, Rouhani and colleagues presented memory items (i.e., scene pictures) at the time of reward prediction as well as reward outcome. In our study, however, images were presented only at the reward-predictive stage, such that feedback RPEs were not temporally coupled with the item to be encoded.

Taken together, our computational analyses suggest similarities in the influence of RPE signaling during experiential and observational decision-making and learning on episodic memory, and further highlight the relevance of the timing of experienced and vicarious RPEs relative to the memory item presentation.

Nonetheless, we acknowledge that the absence of a behavioral measure to assess participants’ predictions and reasoning in observational trials presents a limitation of the current study. To better understand the computational processes involved in observational learning, future studies should consider including such a measure, for example, by asking participants to predict the choice or outcome in observational trials. Additionally, incorporating eye-tracking to analyze gaze patterns and pupil diameter would help assess attention and its role in memory formation. By analyzing gaze behavior, future studies could determine whether participants attend to relevant information equally across both learning conditions. This would allow the assessment of the role of attention during experiential and observational learning on memory formation.

Finally, studies investigating the role of RPEs elicited by one’s own versus others’ behavior in memory formation should consider collecting neural data. Previous research has identified neural markers associated with RPE signaling, feedback processing, and memory retrieval, which could inform computational analyses and contribute to a deeper understanding of how different learning processes influence memory formation.

In summary, our findings highlight the importance of confidence judgments when comparing episodic memory following learning from one’s own versus others’ behavior. While recognition accuracy did not differ between learning conditions, subjective confidence ratings indicated that participants remembered target stimuli more confidently after experiential learning, emphasizing its benefit for conscious recollection. Moreover, both experienced and observed risk-taking (i.e., gambling for potential rewards) enhanced memory accuracy and memory strength. Additionally, we demonstrate a comparable and temporally precise role of experienced and vicarious RPE signaling in memory encoding. Specifically, positive image RPEs were linked to enhanced memory when gambling. Overall, these findings provide evidence for shared mechanisms in experiential and observational decision-making and reward-learning, with similar effects on memory formation, despite stronger subjective memory when learning from one’s own experiences.

## Methods

### Participants

In this study, we collected data from 86 young adults (57 females) between 18 and 30 years old. The sample size was based on a power analysis (α = 0.05, power = 0.8) to reproduce the enhancing effect of positive reward prediction errors on recognition memory (*d* = 0.31) revealed by Jang et al.^26^. We excluded four participants due to too many missing responses (>3 SD from the group mean) and two due to not reaching the performance criterion for the learning task: following Jang et al.^26^, we determined the above-chance performance by simulating random choices using the same learning task structure and calculating the total score achieved. After repeating the simulation 5000 times, we checked whether the participant’s score exceeded 5% of the simulations’ overall score distribution. One additional participant was excluded due to extremely low memory performance ( >3 SD from the group mean), which deviated from an otherwise normal distribution of memory scores across participants. The exclusion resulted in a final sample size of 79 participants (52 females) with an average age of 23.80 ± 4.12 years (mean ± SD) for data analysis. All study participants had normal-to-corrected vision and reported no neurological, psychological, or motoric disorders. Prior to the experiment, we obtained informed written consent from participants. The study was approved by the Ethics Committee of the Institute of Psychology at the Humboldt University of Berlin (reference number 2022-65). All participants attended one experimental session and were subjected to the same experimental conditions. The experiment took a total of 1.5 hours. Participants received 10€ per hour as compensation plus a bonus of 3€. We used a performance-based monetary bonus as an incentive to motivate participants to perform well during the experiment. However, all participants received the full bonus and were informed about this at the end of the experiment.

### Preregistration

The hypotheses, study design, and planned analyses were preregistered on AsPredicted (https://aspredicted.org/7vs3-mkv3.pdf). Deviations from the preregistered analyses – such as exploratory analyses – are described in the Methods and Results sections. Notably, the preregistered hierarchical regression model analysis was not conducted (for more details, see Supplementary Materials: *Hierarchical regression model*; Supplementary Fig. 8).

### Experimental procedure

The experiment comprised two parts: a learning and decision-making task followed by a memory test (Fig. 1a,b). After completing the practice trials, participants performed a decision-making task, where they learned from their own and another agent’s binary choices and feedback. Participants were told at the beginning of the experiment that another participant had already performed the task and that they were observing this other player’s recorded choices. Hence, although the other player’s outcomes did not directly affect the participant’s score, observing the other player’s choices would help the participant to make informed decisions on their turns. In reality, the observed choices were computer-generated to ensure comparability across subjects. This deception was resolved at the end of the experiment. To simulate human-like choice behavior for the observational trials, we employed a reinforcement learning model that was fit to choice behavior derived from 30 participants who previously completed an analogous decision-making task (for more details, see *Reinforcement learning model fitting*). The choices presented in observational trials were simulated using the same task structures that participants underwent in the study. Immediately after the learning task, participants performed a surprise recognition memory test on stimuli seen in the learning task. Their goal in both tasks was to collect as many points as possible by making binary choices.

### Learning task

The learning task consisted of 160 trials with interleaved experiential and observational learning trials (80 trials per learning condition, where no more than two trials of the same condition were presented in a row). The participant’s goal was to learn stimulus-outcome contingencies based on trial-to-trial feedback. On each trial, they either made a binary choice (experiential learning) or observed such a choice by another player (observational learning).

Each trial consisted of four phases: the player, value, image, and feedback phase (Fig. 1a). At the beginning of a given trial, the learning condition was indicated by a cue (“You” – experiential learning, “Other” – observational learning). After an interphase interval (IPI) of 0.5 s (blank screen), a value was displayed. This value was equally sampled from [1, 5, 10, 20, 100] and represented the potential reward of the current trial. Following an IPI of 0.5 s, an image was shown for 3 s, which was assigned to one of two possible categories: animate (e.g., mammals, birds, insects) versus inanimate (e.g., furniture, clothes, food). Each image was unique, so there were never two images of the same animal or object. The two image categories represented opposing reward probabilities (e.g., if the animate category had a reward probability of 80%, the inanimate category had a reward probability of 20%). Participants gave responses during this image presentation phase, which depended on the trial condition. In experiential learning (EL) trials, participants made a play-or-pass choice by pressing the left or right arrow key. The position (left or right) of “Play” and “Pass” on the screen was randomized across participants. Conversely, in the observational learning (OL) trials, participants observed the play-or-pass choice of another player. To ensure participants’ attention, they had to press the spacebar during the image display in order to see the other player’s choice. The chosen option in both conditions was highlighted in black. If participants did not respond within the image presentation time window, a prompt stating that they were too slow appeared, and the next trial started immediately. After an IPI of 0.5 s, the feedback showing the outcome of the current trial was presented for 1.5 s. In this phase, participants, or the other player in OL trials, were rewarded according to the current reward probability. Each trial had an assigned reward probability associated with the given image category. These reward probabilities could change throughout the learning task to a random value between 0 and 1 while always staying oppositely linked (for an example learning task structure, see Supplementary Fig. 9a). The likelihood of a reward probability change was 0.16 on each trial. To enable learning, no changes occurred during the first 20 trials and 15 trials following a probability change. If participants chose to play and the trial was rewarding, they received the points shown in the value phase. If the trial was not rewarding, they always lost 10 points. After choosing to pass, they neither earned nor lost points. Instead, the hypothetical outcome (outcome for a play decision) was presented in brackets. In the OL condition, participants observed the outcome for the other player, which was generated in the same way as in the EL trials. Notably, the observed feedback did not affect the participant’s own score. Following another IPI of 0.5 s, the current cumulative score was displayed for 1 s. Notably, the current score was always and exclusively updated for the current player (EL – participant’s score, OL – other player’s score). The order of trial values and images was pseudorandomized.

Participants were advised to pay attention to trial values and images throughout the task in order to make informed play-or-pass choices. At the beginning of the experiment, they were introduced to the two image categories (animate: animals, inanimate: objects) and were shown example images for each. They were also informed that the reward probabilities associated with the two categories were inversely linked and would occasionally undergo sudden change throughout the task while maintaining this inverse relationship.

### Memory task

In the recognition memory task (Fig. 1b), participants saw all images from the learning task interleaved with new images. The new images (foils) were different depictions of the same animals or objects seen during the learning task. For each image, participants had to state whether it was old (seen before) or new (not seen before) and then had to rate how confident they were regarding their choice. On each trial, one image at a time was shown, and participants had to state whether it was old or new by pressing the left or right arrow key. The position (left or right) of “Old” and “New” on the screen was randomized across subjects. Participants had 5 s to decide and did not receive feedback on whether their choice was correct. Before the memory test, participants were informed that 5 points would be added to their score for every correct answer and 5 points subtracted for every incorrect answer. Following the old-or-new choice and an IPI of 0.5 s, participants were asked to indicate how confident they were regarding their choice. Confidence was rated on a scale from 1 (guess) to 4 (completely certain) by pressing the corresponding number on the keyboard. Participants had 5 s to respond. If they did not make the old-or-new choice or rate their confidence within the time window, a prompt indicating that they were too slow was shown, and the next trial started immediately. The task consisted of 320 trials, with 160 old and 160 new images in pseudorandomized order.

### Debriefing questionnaire

At the end of the experiment, participants filled in a questionnaire concerning both tasks (see Supplementary Materials: *Debriefing questionnaire*). Participants indicated how well they and the other player performed during the learning task, whether they complied with and benefited from the observed choices, and how they felt when the other player received a good or bad outcome, on a Likert scale from 1 to 5.

### Behavioral data analysis

We used the expected reward probability estimated by the Bayesian ideal observer model (see *Bayesian ideal observer model*) when including reward probability in our statistical analyses, to account for changes in reward expectation from the participant’s perspective. All reported mixed-effects linear models (lme4 package^51^, R) were hypothesis-driven and derived from model comparison. The best-fitting model (lowest BIC) was then used for the data analysis. If the models included *learning condition* (EL, OL), *choice* (play, pass), *confidence bin* (low, high), or *attitude* (competitive, non-competitive) as predictors, they were treated as fixed effects and factors. Otherwise, model predictors were scaled and mean-centered.

Participants’ gambling behavior in experiential learning trials was analyzed using a generalized linear mixed-effects model. Based on single-trial data, we modeled the decision to gamble (play) using the within-subject predictors *trial value*, *expected reward probability* ($\exp P({rew})$), and their interaction:

1 $${play} \sim {trial}\,{value}* \exp P({rew})+(1+{trial}\,{value|subject}\,{ID})$$

The predictor *trial value* was treated as a fixed and random effect, while $\exp P({rew})$ was only treated as a fixed effect.

To ensure that the choice behavior in observational learning trials was comparable to the participants’ choice behavior, we analyzed the model-derived choices presented in the OL condition. In line with the participant’s behavior, the proportion of choosing “Play” increased as a function of trial value and expected reward probability (Supplementary Fig. 10a). To compare participants’ and model-derived choice behavior, we combined trial value and reward probability to the expected value. The expected value was estimated per trial based on the magnitude and probability of being rewarded or punished:

2 $${expected}\,{value}={trial}\,{value}* \exp P\left({rew}\right)+\left(-10\right)* \left(1-\exp P\left({rew}\right)\right)$$

We compared participants’ play probability (EL) with the play probability simulated for the same trials (EL) across expected values (Supplementary Fig. 10b), which revealed no evidence of a difference between human behavior and model simulation(independent two-sample t-test; *t*_1262_ = 0.83, *p* = 0.41, *d* = 0.05, 95% CI = −0.02−0.05). This indicated that the observed behavior reflected human choice behavior, thus making the choices in the OL condition credible. Further, it justified computing underlying processes during experiential and observational learning in the same way, and then comparing their effect on subsequent memory.

Participants’ memory performance was first assessed by examining how well they identified old and new images by calculating the discrimination index *d prime* (*d’*; *Z(hit rate) – Z(false-alarm rate)*) per learning condition and participant. Using a one-sample t-test per learning condition, we checked if *d’* values differed from zero to validate that participants’ accuracy was better than chance. The results showed an above-chance accuracy in both learning conditions (Supplementary Fig. 11; EL: mean ± SEM; *d’* = 1.08 ± 0.05, *t*_78_ = 23.59, *p* < 0.001, *d* = 2.65, 95% CI = 0.99-1.17; OL: mean ± SEM; *d’* = 1.03 ± 0.04, *t*_78_ = 23.46, *p* < 0.001, *d* = 2.64, 95% CI = 0.94-1.12), suggesting that participants formed memories in both learning conditions and justifying comparing the two.

Further, we investigated the effect of the learning condition and choice behavior on memory accuracy by estimating a *d’* value per condition (EL play, EL pass, OL play, OL pass) and participant. We ran a linear mixed-effects model on *d’* values with the within-subject predictors *learning condition* (EL, OL)*, choice* (play, pass), and their interaction:

3 $${d}^{{\prime} } \sim {learning}\,{condition}* {choice}+(1{|subject}\,{ID})$$

We applied the same linear mixed-effects model to mean hit rates and false-alarm rates per condition and participant to assess the effect of learning condition and choice behavior on these parameters.

Besides, we examined if memory accuracy was better for higher confidence (confidence rating 3 or 4) than lower confidence (confidence rating 1 or 2) by computing a linear mixed-effects model on *d’* values with the within-subject predictor *learning condition* (EL, OL)*, confidence bin* (low, high), and their interaction:

4 $${d}^{{\prime} } \sim \,{learning}\,{condition}* {confidence}\,{bin}+(1{|subject}\,{ID})$$

In the next step, we considered subjective confidence ratings by combining the old-or-new response and the confidence rating (scale of 1 to 4) per trial into a memory score ranging from 1 to 8 (1 – new and highest confidence, 8 – old and highest confidence). To evaluate whether this score reflects a reasonable measure of subjective memory strength, we checked if this score revealed a positive and roughly linear relationship with the true proportion of old images (Supplementary Fig. 12). Afterwards, we used the memory score as a measure of memory performance in the following analyses. Notably, we focused on the analysis of memory for old images (seen before).

The effect of learning condition and choice behavior on memory was analyzed using a linear mixed-effects model, modeling memory scores with the within-subject predictors *learning condition* (EL, OL), *choice* (play, pass), and their interaction:

5 $${memory}\,{score} \sim {learning}\,{condition}* {choice}+(1{|subject}\,{ID})$$

In addition, we analyzed participants’ responses to the debriefing questionnaire. Participants’ evaluation of their own and the other player’s performance, their compliance with the other player’s choice behavior, and their feelings when witnessing good and bad outcomes for the other player were analyzed using non-parametric tests (i.e., performance, agreement, and happiness). To assess the credibility of our social manipulation, we examined the participants’ perception of the other player in a debriefing questionnaire. Most participants reported that they benefited (75%) from observing the other player and tended to comply (63%) with the observed choices on a 5-point Likert scale (*p*s < 0.001, Wilcoxon signed-rank tests against 3; Supplementary Fig. 13a). Further, participants evaluated their own performance during the learning task similarly to the performance of the other player (*p* = 0.21, Wilcoxon signed-rank test for paired difference; Supplementary Fig. 13b).

In an explorative analysis, we examined the effect of participants’ attitudes toward the other player on memory. Therefore, we analyzed participants’ reported feelings when witnessing good and bad outcomes for the other player. Previous research provided evidence for inverse RPE coding for a confederate’s outcomes, suggesting social comparison even if the task was not competitive and the confederate’s behavior had no effect on the participant’s score^8,52^. We assumed that a competitive attitude would be reflected by being rather happy (rating above 3 – neutral) when the other player received a bad outcome (losing points) and by being rather unhappy (rating below 3 – neutral) when this player obtained a good outcome (earned points). To assess participants’ attitudes, we subtracted the ratings for good outcomes from those for bad outcomes, resulting in one difference score per participant. If the difference score was negative or zero, we assumed this to reflect a non-competitive attitude. If this score was positive, we presumed this to indicate a competitive attitude. Participants were then accordingly coded as competitive or non-competitive. The effect of the competitive attitude on memory was assessed for each learning condition using a linear mixed-effects model. We modeled memory scores (old) per learning condition with the between-subject predictors *attitude* (competitive, non-competitive) and within-subject predictor *choice* (play, pass), and their interaction:

6 $${memory}\,{score} \sim {attitude}* {choice}+(1{|subject}\,{ID})$$

### Computation of reward prediction errors

Reinforcement learning and decision processes can be captured via prediction errors (PEs), which reflect the extent to which an experienced outcome deviates from the predicted outcome^14^. The prediction error indicates whether behavior needs to be adjusted to adapt to changes in the environment and thus is a prerequisite for learning^53^. Reward prediction errors (RPEs) signal whether stored information about the value of different experiences needs to be updated. In our learning task, each phase was associated with one RPE. We calculated RPEs for image and feedback presentation in the same way for experiential and observational trials (Fig. 1c, d):

The *image RPE* resulted from the magnitude and probability of reward (Fig. 1c) and was computed as the difference between the predicted reward before and after the image display (Fig. 1d). Following Jang et al.^26^, the predicted reward on a given trial was calculated for each image category (animate, inanimate) as follows:

7 $${V}_{{pred}}={V}_{{rew}\,}{P}_{{rew}}+{V}_{{pun}}{P}_{{pun}}$$

where the predicted reward (${V}_{{pred}}$) stems from the likelihood (${P}_{{rew}}$) and value (${V}_{{rew}}$) of reward as well as the likelihood $({P}_{{pun}}$) and value (${V}_{{pun}};{\rm{fixed\; at}}-10$) of punishment (Fig. 1d). We assumed the predicted reward before the image presentation was the average across the two image categories. The image RPE was then calculated by subtracting the average reward prediction $({\mu V}_{{pred}})$ from the reward prediction for the displayed image category (${V}_{{pred\_thisCat}}$):

8 $${\mu V}_{{pred}}=({V}_{{pred}\_{thisCat}}\left(t\right)+{V}_{{pred}\_{otherCat}}(t))/2$$

9 $${image}\,{RPE}={V}_{{pred}\_{thisCat}}(t)-{\mu V}_{{pred}}(t)$$

The *feedback RPE* depended on the possible trial outcomes (winning, losing; Fig. 1c) and was computed as the difference between the received outcome $(R\left(t\right))$ and the predicted reward for the shown image category (Fig. 1d):

10 $${feedback}\,{RPE}=R\left(t\right)-{V}_{{pred}\_{thisCat}}(t)$$

Of note, feedback RPE was zero when participants chose to pass.

### Bayesian ideal observer model

We used a Bayesian ideal observer model to estimate the likelihood of a binary outcome (reward, no reward) for each trial while considering changes in the reward probability (change point, CP) in line with Jang et al.^26^. Importantly, although our task introduced observational learning trials, participants received information about trial outcomes regardless of the learning condition. Therefore, we applied the same Bayesian ideal observer model as Jang et al.^26^ to our task to compute the expected reward rate, ideal learning rate, surprise, and uncertainty, as described below (for example task structure, see Supplementary Fig. 9a–e).

As input for the model, we provided information about the binary outcome and the true probability of a change point (*H*; hazard rate) occurring for each trial. The hazard rate was computed per participant by dividing the number of change points by the total number of trials and then used to sample a change point for each trial according to a Bernoulli distribution. When no change in the reward probability occurred, the expected reward rate $({\mu }_{t})$ was updated from the previous trial $({\mu }_{t-1})$. When a change did occur, ${\mu }_{t}$ was set to a random number sampled from a uniform distribution ranging from 0 to 1.

The posterior probability of the expected reward rate ($P({\mu }_{t}|{R}_{1:t})$) was computed as follows:

11 $$P({\mu }_{t}|{R}_{1:t})\propto P({R}_{t}|{\mu }_{t})\mathop{\sum }\limits_{{{CP}}_{t}}\mathop{\sum }\limits_{{\mu }_{t-1}}P({\mu }_{t}|{{CP}}_{t},{\mu }_{t-1})P\left({{CP}}_{t}\right)\,P({\mu }_{t-1}|{R}_{1:t})P({R}_{1:t-1})$$

Here, $P({R}_{t}|{\mu }_{t})$ is the likelihood of the binary outcome based on the expected reward rate, $P({\mu }_{t}|{{CP}}_{t},{\mu }_{t-1})$ is the probability of the reward rate while considering the occurrence of change points, $P\left({{CP}}_{t}\right)$ is the hazard rate, and $P({\mu }_{t-1}|{R}_{1:t})$ is the prior belief of the expected reward rate.

Based on the model-derived expected reward rate, we assessed prediction updates based on the trial outcome and calculated the trial-by-trial ideal learning rate:

12 $$V\left(t+1\right)=V\left(t\right)+\alpha {PE}(t)$$

13 $${PE}=R\left(t\right)-V(t)$$

14 $$\alpha =\frac{V\left(t+1\right)-V(t)}{R\left(t\right)-V(t)}$$

The current belief about the reward rate ($V\left(t+1\right)$) resulted from the previous reward rate belief ($V(t)$), the ideal learning rate $\alpha ,$ and the prediction error (${PE}$). The PE was computed as the difference between the obtained ($R(t)$) and predicted outcomes ($V\left(t\right)$).

Using the Bayesian ideal observer model, we further calculated surprise and uncertainty for each trial to consider their potential effect during reinforcement learning on memory formation. Surprise ($P({{CP}}_{t}|{R}_{1:t})$) indicated the likelihood that a change point had occurred on a given trial based on previous outcome observations. This trial-by-trial modulation of change-point probability was computed by marginalizing the expected reward rate:

15 $$P({{CP}}_{t}|{R}_{1:t})\propto P({R}_{t}|{\mu }_{t})\mathop{\sum }\limits_{{\mu }_{t}}\mathop{\sum }\limits_{{\mu }_{t-1}}P({\mu }_{t}|{{CP}}_{t},{\mu }_{t-1})P\left({{CP}}_{t}\right)P({\mu }_{t-1}|{R}_{1:t})P({R}_{1:t-1})$$

Uncertainty ($U\left(t\right)$) was estimated by calculating the entropy of the posterior probability distribution of the reward rate for each trial:

16 $$U\left(t\right)=-\sum P({\mu }_{t}{{|R}}_{1:t})\mathrm{ln}(P({\mu }_{t}|{R}_{1:t}))$$

### Reward prediction errors and memory

To assess the impact of reward-related computational parameters (i.e., trial value, RPEs, surprise, and uncertainty) on memory, we fitted linear regression models for each parameter of interest. Specifically, we were interested in the relationship between memory scores and RPEs (i.e., image RPE, feedback RPE). Memory scores were mean-centered, and predictors were z-score normalized before fitting a linear regression model per condition (EL play, EL pass, OL play, OL pass) and participant. One-sample t-tests against zero were then applied to the resulting slope estimates to test for the overall effect of each parameter on memory across participants. To account for multiple comparisons, we applied Bonferroni corrections to the α level. We next examined whether the relationship between memory and each computational parameter was affected by learning condition or choice behavior. Therefore, we modeled regression slopes using linear mixed-effects models with the within-subject predictors *learning condition* (IL, OL), *choice* (play, pass), and their interaction:

17 $${slope}\, \sim {learning}\,{condition}* {choice}+(1{|subject}\,{ID})$$

We examined the influence of more extreme trial values (i.e., values deviating from the average expected trial value of 10) on memory. To do so, trial values were grouped in three bins (bin 1: 10, bin 2: 5 and 20, bin 3: 1 and 100), before fitting linear regression models to these bins and mean memory scores per value bin. The resulting slopes were analyzed using a linear regression model (lm package, R):

18 $${slope}\, \sim {learning}\,{condition}* {choice}$$

due to the singular fit of the mixed-effects model, including the subject as a random effect, indicating negligible variation in participants’ intercepts. Consequently, we removed the random effect.

As previous research has highlighted the memory-enhancing effect of positive RPEs^26,28^, we were mainly interested in the influence of positive image RPEs. In addition, we observed that while a wide range of image RPEs were represented per participant, the number of trials with a high RPE magnitude (e.g., > 30 or < -30) was disproportionately low when compared to trials with a lower magnitude (Supplementary Fig. 14). To account for this and to increase the robustness of our analysis, we therefore focused on the effect of positive and negative RPEs. To this end, image RPEs were binned by sign (bin 1: RPE < 0, bin 2: RPE > 0) before fitting linear regression models to binned RPEs and mean memory scores per RPE bin. For comparison, slope analyses using continuous image RPEs are reported in Supplementary Materials (Supplementary Table 21). In an exploratory analysis, we assessed memory performance separately for positive and negative image RPE trials. We examined if memory scores in ‘play’ (respectively ‘pass’) trials eliciting positive (respectively negative) RPEs differed from average by computing a t-test against zero on mean-centered memory scores.

We further analyzed the relationship between participants’ sensitivity to trial information that shape image RPEs and their effect on memory. For each participant, we fit a logistic regression model predicting choices (play, pass) from *trial value* and *expected reward probability*. Additionally, we fit a linear regression model to memory scores and binned image RPEs. For all regressions, memory scores were mean-centered, and predictors were z-score normalized. Finally, we computed a Spearman correlation between the slope estimates derived from the logistic and linear regression.

To maintain consistency with the image RPE analysis, feedback RPEs were also binned by sign (bin 1: RPE < 0, bin 2: RPE > 0), before fitting a linear regression model to these bins and mean memory scores per RPE bin. Slope analyses using continuous feedback RPEs are reported in Supplementary Materials (Supplementary Table 22). In an exploratory analysis, we analyzed the effect of the absolute magnitude of these continuous feedback RPEs (unsigned RPEs) on memory.

### Reinforcement learning model fitting

We applied various reinforcement learning (RL) models directly to participants’ choice behavior to investigate the role of learning-related computational parameters in memory encoding. Parameter estimation was conducted using a constrained optimization algorithm (*fmincon* in MATLAB; see Jang et al.^26^) which identified the set of parameters that maximized the total log-posterior probability of the observed choice data. Importantly, although the models incorporated latent variables spanning all trials, including both EL and OL conditions, parameter fitting was restricted exclusively to the choice behaviors during experiential learning trials.

Our base model contained four free parameters: (1) a temperature parameter (inverse T) of the softmax function, (2) a value exponent term scaling the effect of trial value on choice behavior, (3) a play bias term reflecting the attribution of higher value to play decisions, and (4) an intercept term that scales the effect of learning rate on choice behavior. The second and third parameters accounted for possible interindividual differences in choice behavior due to biases towards certain trial features^26^. Participants might attribute different values to the potential payout (trial value) and to trials in which they decided to gamble (play). Combining these potential influences on choice behavior, we computed an overall *biased value* on each trial:

19 $${V}_{B}\left(t\right)={B}_{{play}}+({P}_{{rew}}\times {V}_{t}^{k}+(1-{P}_{{rew}})\times {(-10)}^{k})$$

Here, ${B}_{{play}}$ is the play bias term, ${V}_{t}$ is the trial value presented at the beginning of the trial and $k$ is the value exponent. We then used the softmax function to convert this overall ‘biased’ value into the likelihood to play ($P({play|}{V}_{B}\left(t\right))$) or to pass ($P({pass|}{V}_{B}\left(t\right))$).

We fit the described base model and additional RL models to the participants’ choice behavior by adding parameters to our base model. These additional parameters controlled the extent to which other task-relevant variables affected the trial-to-trial modulation of the learning rate, including surprise, uncertainty, and the ideal learning rate computed from the Bayesian ideal observer model. Specifically, the learning rate was determined by a logistic function of a weighted predictor matrix that included the above variables and an intercept term. Thus, the model captured the degree to which the learning rate changed as a function of these variables. For example, for the model including surprise and uncertainty, the learning rate was updated as follows:

20 $${\alpha }_{l}=\frac{\exp \left({x}_{\alpha }\right)}{1+\exp \left({x}_{\alpha }\right)}$$

21 $${x}_{\alpha }={\beta }_{0}+{\beta }_{1}* P({{CP}}_{t}|{R}_{1:t})+{\beta }_{2}* U\left(t\right)$$

where ${\beta }_{0}$ is the intercept term, ${\beta }_{1}$ is the parameter for surprise ($P({{CP}}_{t}|{R}_{1:t})$), and ${\beta }_{2}$ is the parameter for uncertainty ($U\left(t\right)$). Building on the findings of Jang et al.^26^, we hypothesized that participants’ choice behavior would vary depending on whether trial information was derived from the EL and OL conditions. Specifically, we posited that participants might adjust their learning rate differently in response to trial feedback obtained during the observational trials. To test this hypothesis, we extended our analysis by fitting additional RL models in which the parameter estimates were computed separately for each learning condition ($l\in \{{IL},{OL}\}$), such that

22 $${\alpha }_{l}=\frac{\exp \left({x}_{\alpha ,l}\right)}{1+\exp \left({x}_{\alpha ,l}\right)}$$

23 $${x}_{\alpha ,l}={\beta }_{0,l}+{\beta }_{1,l}* P({{CP}}_{t}|{R}_{1:t})+{\beta }_{2,l}* U\left(t\right)$$

Given the large number of potential models to consider, we carried out parameter and model recovery analyses to refine the model set considered in our final analysis (see Supplementary Materials: *Reinforcement learning model fitting and selection*, Supplementary Figs. 1,2). The eight models presented in the main results are a result of this model selection process. From these, the best-fitting model was determined by computing the Akaike information criterion (AIC) for each model and then comparing these values to that of the base model. Further, we assessed the fit of our winning model by comparing participants’ choice behavior to the model-predicted behavior.

Following, we checked whether the split parameter estimates, surprise and uncertainty, from the best-fitting model significantly affected the learning rate and further whether they differed between learning conditions using non-parametric tests. Moreover, we examined the link between the parameter estimates and memory while controlling for an effect of the learning condition. Here, we modeled surprise and uncertainty estimates each using a robust linear regression model (robustlmm package, R), including the within-subject predictor *learning condition* (IL, OL) and *memory score* (old) as well as their interaction term.

### Simulation of observed choice behavior

To simulate realistic, human-like choice behavior for the OL condition, we fitted two RL models – our base (as described above) model and an extended model^26^ – directly to the choice behavior from 30 participants who conducted 80 experiential learning trials (Supplementary Fig. 15).

The extended model contained the same free parameters as our base model and two additional ones, surprise and uncertainty, which contributed to the trial-to-trial modulation of the learning rate. Following, we assessed model fits by computing the Bayesian information criterion (BIC) for each model and then comparing them. Since BIC values indicated that both models performed similarly well, we chose the simpler model (base model) to generate the choices for the OL condition. Here, we used the model-derived expected trial value (${V}_{B}\left(t\right)$) and the temperature parameter estimates as inputs to the softmax function to generate a choice for each trial.

## Supplementary information

Supplementary Materials

## Data Availability

All data from this study are publicly available on the Open Science Framework (OSF; https://osf.io/fsbzd/).

## Code availability

All analysis scripts and experimental programs are publicly available on the Open Science Framework (OSF; https://osf.io/fsbzd/). Data analysis was performed using MATLAB (Version R2024b, MathWorks, 2024) and R (Version 4.3.3, R Core Team, 2024).
