# Supplementary material for: Shared effects of one’s own and others’ experiences during reinforcement learning on episodic memory
Source: NPJ Sci Learn. 2026 Feb 28;11:16. doi: 10.1038/s41539-026-00409-7 (PMC12992901; doi:10.1038/s41539-026-00409-7)
Supplement: Supplementary file 1 — Supplementary Materials [file 41539_2026_409_MOESM1_ESM.pdf]

Supplementary Materials for

Shared effects of one's own and others' experiences during reinforcement learning on episodic memory

Short title: **Memory following experiential and observational reinforcement learning**

Maria A. Woitow[1]*, Anthony I. Jang[2], Ben Eppinger[3,4], Matthew R. Nassar[5,6], Marcel Brass[1,7,8] & Julia M. Rodriguez Buritica[1,3,7]

[1]Berlin School of Mind and Brain & Department of Psychology, Humboldt-Universität zu Berlin, Berlin, Germany; [2]Department of Psychiatry and Biobehavioral Sciences, Jane and Terry Semel Institute for Neuroscience and Human Behavior, University of California Los Angeles, Los Angeles, USA; [3]Department of Psychology, University of Greifswald, Greifswald, Germany; [4]Department of Psychology, Concordia University, Montreal, Canada; [5]Robert J. and Nancy D. Carney Institute for Brain Science, Brown University, Providence, USA; [6]Department of Neuroscience, Brown University, Providence, USA; [7]Science of Intelligence, Research Cluster of Excellence, Berlin, Germany; [8]Department of Experimental Psychology, Ghent University, Belgium.

Corresponding author:

Maria A. Woitow
Mail: maria.woitow@hu-berlin.de
Address: Social Intelligence Lab, Department of Psychology, Humboldt-Universität zu Berlin, Luisenstraβe 56, Haus 5, 10115 Berlin, Germany

Copyright
All Figures presented in the Supplementary Materials are the original work of the authors.

This PDF file includes:

Supplementary Tables 1 to 22
Supplementary Figures 1 to 15
Debriefing questionnaire
Supplementary Reference

Contents

Participants' gambling behavior 4

Supplementary Table 1 4

Reinforcement learning model fitting and selection..4

Parameter recovery..4

Supplementary Figure 1 5

Model recovery..6

Supplementary Figure 2 6

Memory accuracy 7

Supplementary Table 2..7

Supplementary Figure 3 7

Supplementary Table 3..8

Supplementary Table 4..8

Supplementary Table 5..8

Memory strength 8

Supplementary Table 6..8

Effect of competitiveness on memory 9

Supplementary Table 7..9

Supplementary Table 8..9

Effect of trial values on memory..9

Supplementary Table 9..9

Supplementary Figure 4 10

Supplementary Table 10..10

Supplementary Table 11..10

Effect of image RPEs on memory..11

Supplementary Figure 5 11

Supplementary Table 12..11

Supplementary Table 13..11

Supplementary Table 14..12

Supplementary Table 15..12

Effect of feedback RPEs on memory 12

Supplementary Table 16..12

Supplementary Figure 6 13

Supplementary Table 17 13
Effect of surprise and uncertainty on memory 14
Supplementary Figure 7 14
Supplementary Table 18 15
Supplementary Table 19 15
Supplementary Table 20 15
Hierarchical regression model 16
Supplementary Figure 8 16
Learning task structure 17
Supplementary Figure 9 17
Other player's gambling behavior 18
Supplementary Figure 10 18
Memory accuracy 18
Supplementary Figure 11 18
Proportion of old images vs. memory score 19
Supplementary Figure 12 19
Evaluation of participants' and other player's learning task performance 19
Supplementary Figure 13 19
Continuous image RPEs 20
Supplementary Figure 14 20
Supplementary Table 21 20
Continuous feedback RPEs 20
Supplementary Table 22 20
Reinforcement learning model for simulation of other player's gambling behavior 21
Supplementary Figure 15 21
Debriefing questionnaire 22
References 23

Participants’ gambling behavior

Supplementary Table 1: Generalized linear mixed-effects model on the effect of trial value and expected reward probability (expP(rew)) on gambling (play choice)

	β	*SE*	*z*	*p*
Intercept	0.31	0.09	3.56	< 0.001
Trial value	1.35	0.17	8.02	< 0.001
expP(rew)	1.42	0.04	33.49	< 0.001
Trial value x expP(rew)	0.24	0.055	4.37	< 0.001

McFadden’s r-squared = 0.29
AUC = 0.86
Overall accuracy = 78.21%
Sensitivity = 78.77%
Specificity = 77.66%

Reinforcement learning model fitting and selection

To assess the quality of model fits and reduce the number of potential models, we conducted parameter and model recovery analyses. Based on prior work, we considered the following free parameters for model fitting: inverse temperature, value exponent, play bias, the ideal learning rate (LR), the LR intercept term, surprise, and uncertainty. Additional models were fitted after separating the parameters between the EL and OL trials for intercept, surprise, and uncertainty, yielding a total of nine different models (Supplementary Figure 2).

Parameter recovery

To perform parameter recovery of a given model, we first randomly selected a task structure (i.e., sequence of trials and their associated values and outcomes) from those completed by the participants. We then simulated fake data with a new set of parameter values (Simulated Parameters) that were randomly sampled from the range of ‘true’ parameter values from the participants’ choice behavior. The random sampling was done after removing outliers defined as three-scaled mean absolute deviations (MAD) from the median to prevent fitting extreme values. Then, we fitted the model again to these fake data to recover those same parameters (Fit Parameters; see Wilson & Collins[1]). We repeated the above procedure 100 times, each time choosing a new randomly selected task structure. Therefore, for each parameter of a given model, we can plot the simulated vs. fitted parameters and compute their correlation (Supplementary Figure 2). Upon evaluation of these scatterplots, the inverse temperature variable was removed given it did not consistently yield a correlation coefficient of at least 0.5 across all models.

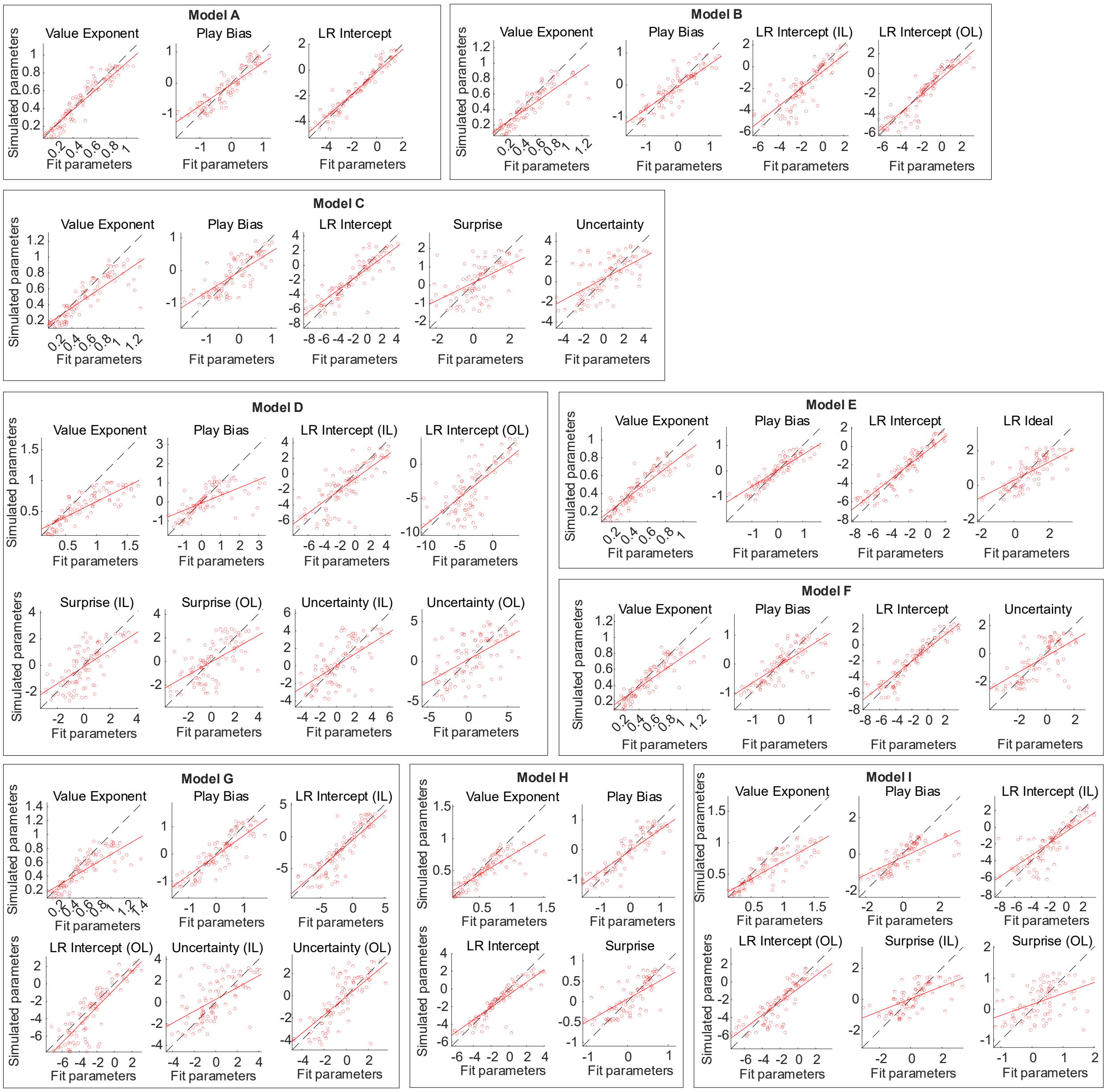

Supplementary Figure 1. Parameter recovery for all nine initial models. Red points represent the simulated and fitted parameters for each random iteration and the red lines show the least-squares line for each scatterplot. The black dotted lines represent the identity line. Models are labeled with alphabetical labels (A-I) to avoid confusion with the numbered system of the eight models shown in the main paper.

Model recovery

Next, we conducted model recovery to evaluate whether each model could be reliably recovered using simulated data[1]. This involved simulating data from all nine models, following the same procedure as in the parameter recovery analysis. We then fitted the simulated data using all models to assess how well the generated data from a given model was identified as the best fitted for that model compared to others. For model selection, we used the Akaike Information Criterion (AIC) over the Bayesian Information Criterion (BIC) to mitigate a concern that BIC may impose a stronger penalty for model complexity, which can disproportionately penalize models with more parameters (i.e., those models that split parameters for the EL and OL conditions). The results are presented in a confusion matrix, which quantifies the probability of each model being identified as the best fitted for data generated by all models (i.e., lowest AIC among all nine models; Supplementary Figure 3). Any models that yielded a lower rate of model recovery compared to other models were excluded, resulting in the removal of Model F.

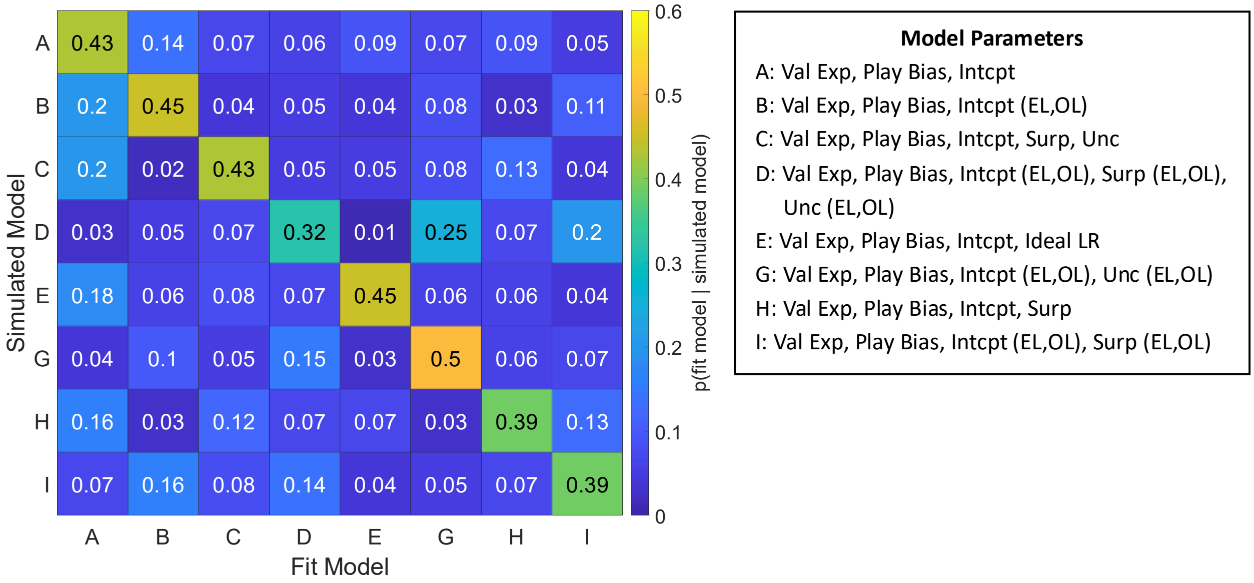

Supplementary Figure 2. Model recovery confusion matrix. The color and corresponding number in each grid represent p(fit model | simulated model).

Memory accuracy

Supplementary Table 2: Linear mixed-effects model on the effect of learning condition (EL, OL) and choice (play, pass) on memory accuracy (*d'*)

	β	***SE***	***df***	***t***	***p***
Intercept	1.07	0.04	78	25.86	< 0.001
Condition – EL	0.03	0.02	234	1.48	0.14
Choice – play	0.04	0.02	234	2.26	0.025
Condition x choice	0.01	0.02	234	0.50	0.62

$R^2c = 0.48$, $R^2m = 0.012$

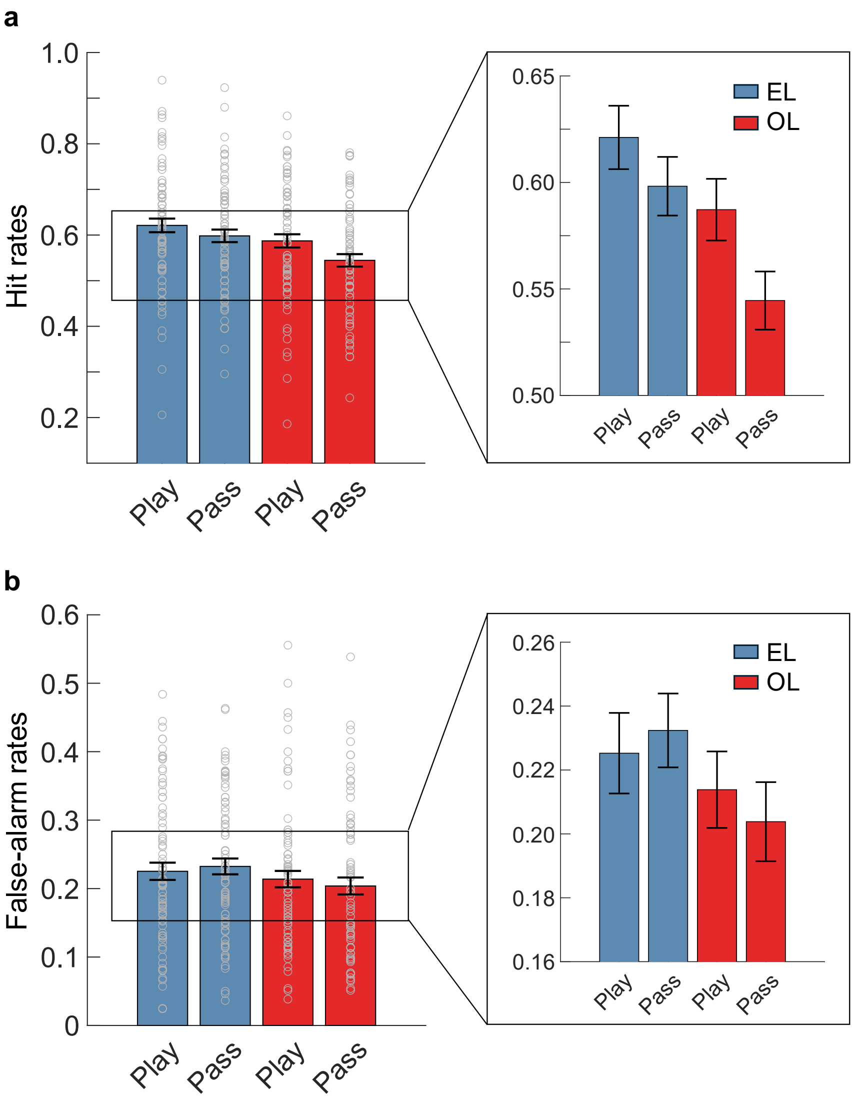

Supplementary Figure 3. Hit rates and false-alarm rates. **a–b.** Plots contain individual data points (each circle represents one participant, n = 79). **a.** Average (mean ± SEM) hit rates per learning condition (EL, OL) and choice (Play, Pass). **b.** Average (mean ± SEM) false-alarm rate per learning condition (EL, OL) and choice (Play, Pass).

Supplementary Table 3: Linear mixed-effects model on the effect of learning condition (EL, OL) and choice (play, pass) on hit rates

	β	*SE*	*df*	*t*	*p*
Intercept	0.59	0.01	78	51.86	< 0.001
Condition – EL	0.02	0.005	234	4.62	< 0.001
Choice – play	0.02	0.005	234	3.47	< 0.001
Condition x choice	-0.005	0.005	234	-1.04	0.30

$R^2c = 0.58$, $R^2m = 0.05$

Supplementary Table 4: Linear mixed-effects model on the effect of learning condition (EL, OL) and choice (play, pass) on false-alarm rates

	β	*SE*	*df*	*t*	*p*
Intercept	0.22	0.01	78	21.05	< 0.001
Condition – EL	0.01	0.004	234	2.76	0.006
Choice – play	0.0007	0.004	234	0.20	0.84
Condition x choice	-0.004	0.004	234	-1.18	0.24

$R^2c = 0.65$, $R^2m = 0.01$

Supplementary Table 5: Linear mixed-effects model on the effect of learning condition (EL, OL) and confidence bin (low confidence, high confidence) on memory accuracy (*d'*)

	β	*SE*	*df*	*t*	*p*
Intercept	0.99	0.04	78	26.06	< 0.001
Condition – EL	0.03	0.02	225	1.20	0.23
Confidence bin – high confidence	0.38	0.02	227	16.86	< 0.001
Condition x conf bin	-0.009	0.02	225	-0.42	0.68

$R^2c = 0.59$, $R^2m = 0.39$

Memory strength

Supplementary Table 6: Linear mixed-effects model on the effect of learning condition (EL, OL) and choice (play, pass) on memory score (old)

	β	*SE*	*df*	*t*	*p*
Intercept	5.12	0.06	78	89.22	< 0.001
Condition – EL	0.12	0.02	234	5.35	< 0.001
Choice – play	0.07	0.02	234	3.10	0.002
Condition x choice	-0.008	0.02	234	-0.34	0.73

$R^2c = 0.60$, $R^2m = 0.05$

Effect of competitiveness on memory

Supplementary Table 7: Linear mixed-effects model on the effect of the attitude (competitive, non-competitive) toward the other player and choice (play, pass) on the memory score (old) in the OL condition. Corrected α = 0.025

	β	***SE***	***df***	***t***	***p***
Intercept	4.99	0.06	77	78.23	< 0.001
Choice – play	0.08	0.03	77	3.21	0.002
Attitude – competitive	0.08	0.06	77	1.17	0.25
Choice x attitude	-0.05	0.03	77	-1.78	0.08

$R^2c = 0.72$, $R^2m = 0.04$

Supplementary Table 8: Linear mixed-effects model on the effect of the attitude (competitive, non-competitive) toward the other player and choice (play, pass) on the memory score (old) in the EL condition. Corrected α = 0.025

	β	***SE***	***df***	***t***	***p***
Intercept	5.22	0.06	77	84.12	< 0.001
Choice – play	0.06	0.03	77	1.98	0.051
Attitude – competitive	0.16	0.06	77	2.50	0.015
Choice x attitude	0.006	0.03	77	0.20	0.85

$R^2c = 0.63$, $R^2m = 0.07$

Effect of trial values on memory

Supplementary Table 9: One-sample t-tests against 0 on the slopes derived from linear regressions fitted to memory scores (old) and trial values per learning condition (EL, OL) and choice (play, pass). Corrected α = 0.0125

Memory ~ trial value	***t***	***df***	***95% CI***	***d***	***p***
EL play	-2.37	78	-0.005 – (-0.0004)	-0.27	0.02
EL pass	0.67	78	-0.004 – 0.008	0.08	0.50
OL play	-0.14	78	-0.002 – 0.002	-0.02	0.89
OL pass	-1.81	78	-0.005 – 0.0003	-0.20	0.07

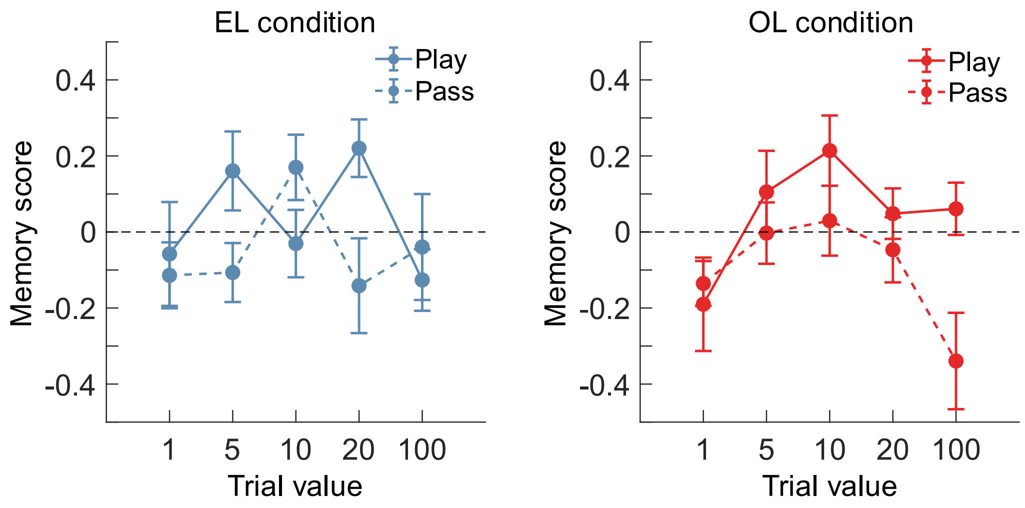

Supplementary Figure 4. Effect of trial value (i.e., signed value RPE) on memory. The slopes were derived from linear regressions fitted to memory scores (old) and trial values per learning condition (EL, OL) and choice (play, pass). The slopes are plotted for each trial value bin (mean ± SEM) per choice (play, pass) separately for each learning condition (EL, OL).

Supplementary Table 10: One-sample t-tests against 0 on the slopes derived from linear regressions fitted to memory scores (old) and more extreme trial values (3 bins: bin 1 - 10, bin 2 - 5 & 20, bin 3 - 1 & 100) per learning condition (EL, OL) and choice (play, pass). Corrected α = 0.0125

Memory ~ trial value	***t***	***df***	***95% CI***	***d***	***p***
EL play	-1.24	78	-0.18 – 0.04	-0.14	0.22
EL pass	-1.88	78	-0.24 – 0.007	-0.21	0.06
OL play	-1.92	78	-0.21 – 0.004	-0.22	0.06
OL pass	-1.93	78	-0.21 – 0.003	-0.22	0.06

Supplementary Table 11: Linear model on effect of learning condition (EL, OL) and choice (play, pass) on the slopes derived from linear regressions fitted to more extreme trial values (3 bins; bin 1: 10 – average value, bin 2: 5, 20 – moderate values, bin 3: 1, 100 – extreme values) and mean memory scores (old) per bin

	β	***SE***	***t***	***p***
Intercept	-0.1	0.028	-3.48	< 0.001
Condition – EL	0.006	0.028	0.21	0.83
Choice – play	0.01	0.028	0.41	0.68
Condition x choice	0.01	0.028	0.45	0.66

$R^2c = 0.001$, $R^2m = -0.008$

Effect of image RPEs on memory

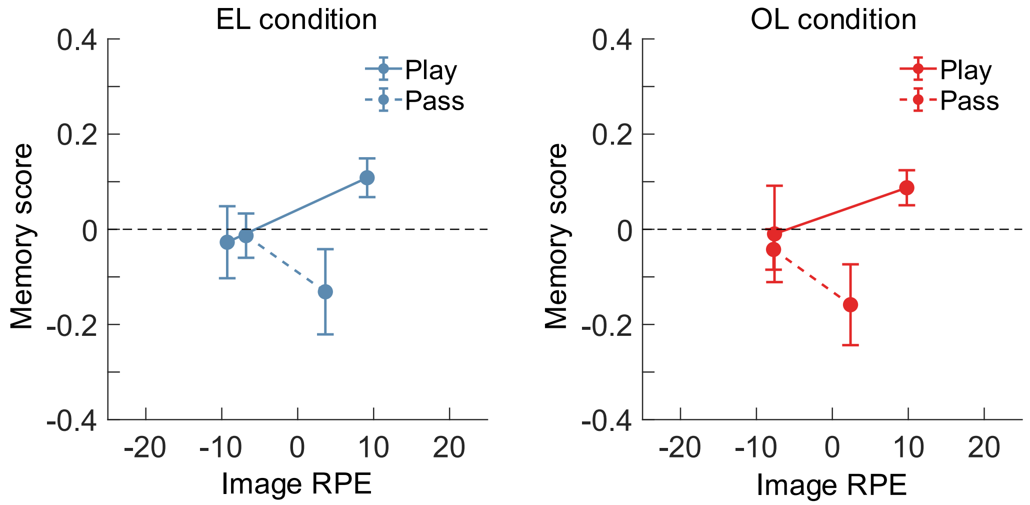

Supplementary Figure 5. Effect of image RPEs on memory scores (old). The slopes derived from linear regressions fitted to binned image RPEs (bin 1: PE < 0, bin 2: PE > 0) and mean memory scores (old) per image RPE bin for each learning condition (EL, OL) and choice (play, pass). The slopes are plotted for each image PE bin (mean ± SEM) per choice (play, pass) separately for each learning condition (EL, OL).

Supplementary Table 12: One-sample t-tests against 0 on the slopes derived from linear regressions fitted to memory scores (old) and binned image RPEs (bin 1: RPE < 0, bin 2: RPE > 0) per learning condition (EL, OL) and choice (play, pass). Corrected α = 0.0125

Memory ~ image RPE	***t***	***df***	***95% CI***	***d***	***p***
EL play	1.33	78	-0.004 – 0.02	0.15	0.19
EL pass	-0.61	78	-0.04 – 0.02	-0.07	0.54
OL play	1.25	78	-0.006 – 0.02	0.14	0.22
OL pass	-1.31	78	-0.04 – 0.009	-0.15	0.20

Supplementary Table 13: Linear mixed-effects model on the effect of learning condition (EL, OL) and choice (play, pass) on the slopes derived from linear regressions fitted to binned image RPE (bin 1: RPE < 0, bin 2: RPE > 0) and mean memory scores (old) per bin

	β	***SE***	***df***	***t***	***p***
Intercept	-0.002	0.006	78	-0.43	0.67
Condition – EL	0.002	0.005	234	0.33	0.74
Choice – play	0.011	0.005	234	2.02	0.045
Condition x choice	-0.003	0.005	234	-0.53	0.60

R^2c = 0.04, R^2m = -0.014

Supplementary Table 14: One-sample t-tests against 0 on mean memory scores (old) for pass trials eliciting positive image RPEs per learning condition (EL, OL). Corrected α = 0.025

Memory	*t*	*df*	*95% CI*	*d*	*p*
EL pass	-1.47	78	-0.31 – 0.05	-0.17	0.15
OL pass	-1.87	78	-0.33 – 0.01	-0.21	0.07

Supplementary Table 15: One-sample t-tests against 0 on mean memory scores (old) for play and pass trials eliciting negative image RPEs per learning condition (EL, OL). Corrected α = 0.025

Memory	*t*	*df*	*95% CI*	*d*	*p*
EL play	-0.36	78	-0.18 – 0 .12	-0.04	0.72
EL pass	-0.29	78	-0.11 – 0.08	-0.03	0.78
OL play	-0.10	78	-0.21 – 0.19	-0.01	0.92
OL pass	-1.00	78	-0.13 – 0.04	-0.11	0.32

Effect of feedback RPEs on memory

Supplementary Table 16: One-sample t-tests against 0 on the slopes derived from linear regressions fitted to memory scores (old) and binned feedback PEs (bin 1: RPE < 0, bin 2: RPE > 0) per learning condition (EL play, OL play) and feedback timing (curr - current, prev - previous). Corrected α = 0.0125

Memory ~ signed feedback PE	*t*	*df*	*95% CI*	*d*	*p*
EL play curr	1.95	78	-0.0001 – 0.008	0.22	0.054
OL play curr	0.64	78	-0.004 – 0.008	0.07	0.52
EL play prev	0.58	78	-0.006 – 0.01	0.07	0.57
OL play prev	0.87	78	-0.004 – 0.01	0.10	0.39

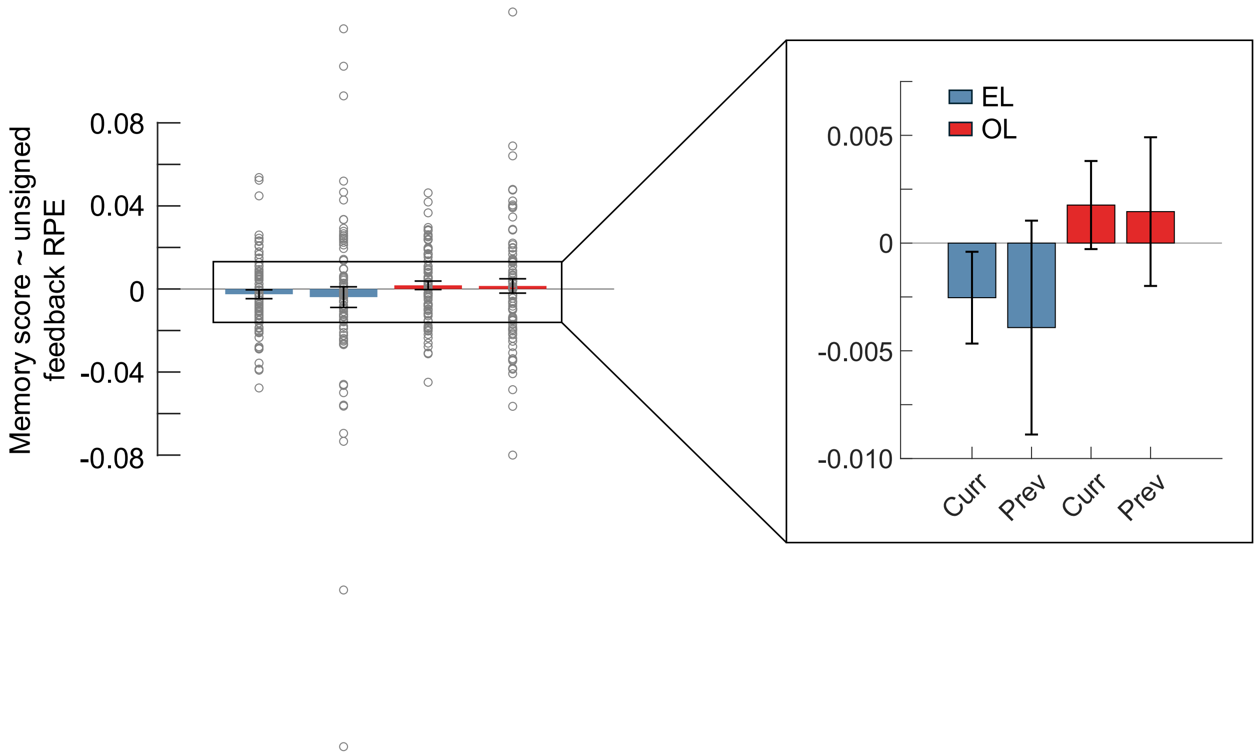

Supplementary Figure 6. Effect of unsigned feedback RPEs on memory. **a.** The average slopes (mean ± SEM) derived from linear regressions fitted to memory scores (old) and unsigned feedback RPEs per learning condition (EL, OL) and trial type (Curr – current, Prev – previous). The trial type distinguishes the effect of the current trial's unsigned feedback RPE (t) on memory for the same trial (t) and the effect of the previous trial's unsigned feedback RPE (t-1) on memory for the subsequent trial (t). Average slopes are plotted with individual data points (each grey circle represents one participant).

Supplementary Table 17: One-sample t-tests against 0 on the slopes derived from linear regressions fitted to memory scores (old) and unsigned feedback PEs (curr – current, prev - previous) per learning condition (EL play, OL play). Corrected α = 0.0125

Memory ~ unsigned feedback PE	***t***	***df***	***95% CI***	***d***	***p***
EL play curr	-1.19	78	-0.007 – 0.002	-0.13	0.24
OL play curr	0.86	78	-0.002 – 0.006	0.10	0.39
EL play prev	-0.79	78	-0.01 – 0.006	-0.09	0.43
OL play prev	0.42	78	-0.005 – 0.008	0.05	0.67

Effect of surprise and uncertainty on memory

a

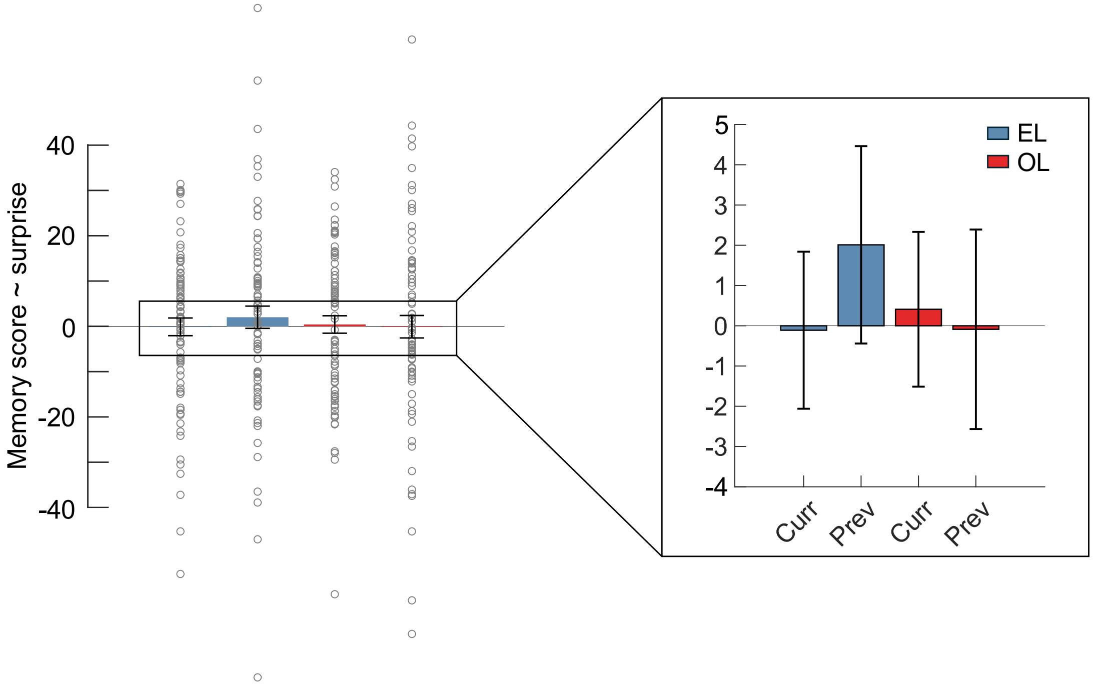

b

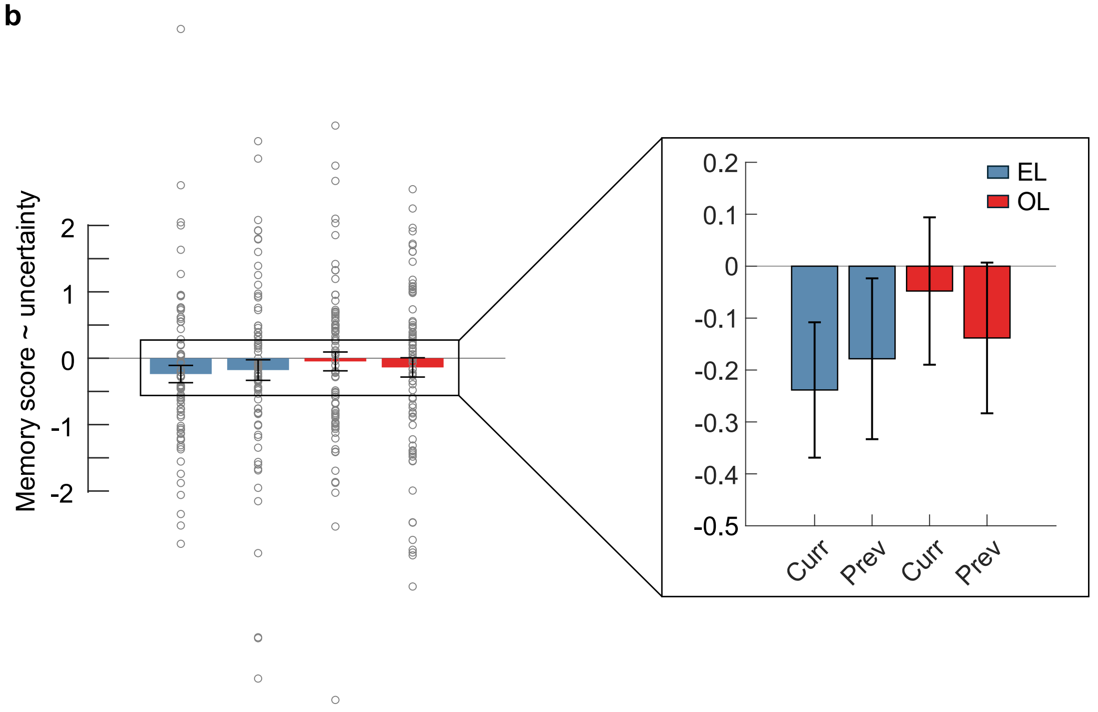

Supplementary Figure 7. Effect of surprise and uncertainty on memory. **a–b.** The average slopes (mean ± SEM) derived from linear regressions fitted to memory scores (old) and **(a)** surprise and **(b)** uncertainty estimates per learning condition (EL, OL) and trial type (Curr – current, Prev – previous). The trial type distinguishes the effect of the current trial's **(a)** surprise (t) and **(b)** uncertainty (t) on memory for the same trial (t) and the effect of the previous trial's **(a)** surprise (t-1) and **(b)** uncertainty (t-1) on memory for the subsequent trial (t). Average slopes are plotted with individual data points (each grey circle represents one participant).

Supplementary Table 18: One-sample t-tests against 0 on the slopes derived from linear regressions fitted to memory score (old) and surprise estimates (curr – current, prev – previous) per learning condition (EL play, OL play). Corrected α = 0.0125

Memory ~ surprise	***t***	***df***	***95% CI***	***d***	***p***
EL play curr	-0.06	78	-3.99 – 3.77	-0.006	0.96
OL play curr	0.21	78	-3.42 – 4.24	0.02	0.83
EL play prev	0.82	78	-2.87 – 6.89	0.09	0.42
OL play prev	-0.04	78	-5.02 – 4.85	-0.004	0.97

Supplementary Table 19: One-sample t-tests against 0 on the slopes derived from linear regressions fitted to memory score (old) and uncertainty estimates (curr – current, prev – previous) per learning condition (EL play, OL play). Corrected α = 0.0125

Memory ~ uncertainty	***t***	***df***	***95% CI***	***d***	***p***
EL play curr	-1.83	78	-0.50 – 0.02	-0.21	0.07
OL play curr	-0.34	78	-0.33 – 0.24	-0.04	0.74
EL play prev	-1.15	78	-0.49 – 0.13	-0.13	0.25
OL play prev	-0.95	78	-0.43 – 0.15	-0.11	0.34

Supplementary Table 20: Paired-samples t-tests for learning condition difference on slope between memory score and surprise/ uncertainty estimates (curr – current, prev – previous). Corrected α = 0.025

Difference EL vs. OL	***t***	***df***	***95% CI***	***d***	***p***
Surprise curr	-0.20	78	-5.73 – 4.69	-0.02	0.84
Surprise prev	0.58	78	-5.12 – 9.32	0.07	0.56
Uncertainty curr	-0.97	78	-0.58 – 0.20	-0.11	0.34
Uncertainty prev	-0.20	78	-0.44 – 0.36	-0.02	0.84

Hierarchical regression model

As outlined in our study's pre-registration, we planned to construct a hierarchical regression model to pool information across participants and account for multiple sources of variability, such as participant- and stimulus-specific effects. Before implementing this, we first investigated whether this approach was likely to provide additional insights not captured by simpler linear regression models. To do so, we constructed a regression model that included additional categorical variables for each participant and each image's unique identifier and category (i.e., animate vs. inanimate), then examined whether this inclusion affected the associations between memory score and our variables of interest, such as image RPE, reward probability, surprise, and uncertainty. The mean regression weights for each of these variables remained qualitatively similar and did not change significantly with the addition of these categorical variables (Supplementary Figure 15). Therefore, we concluded that a hierarchical regression model was unlikely to yield additional insights on any participant- or stimulus-specific effects in our data.

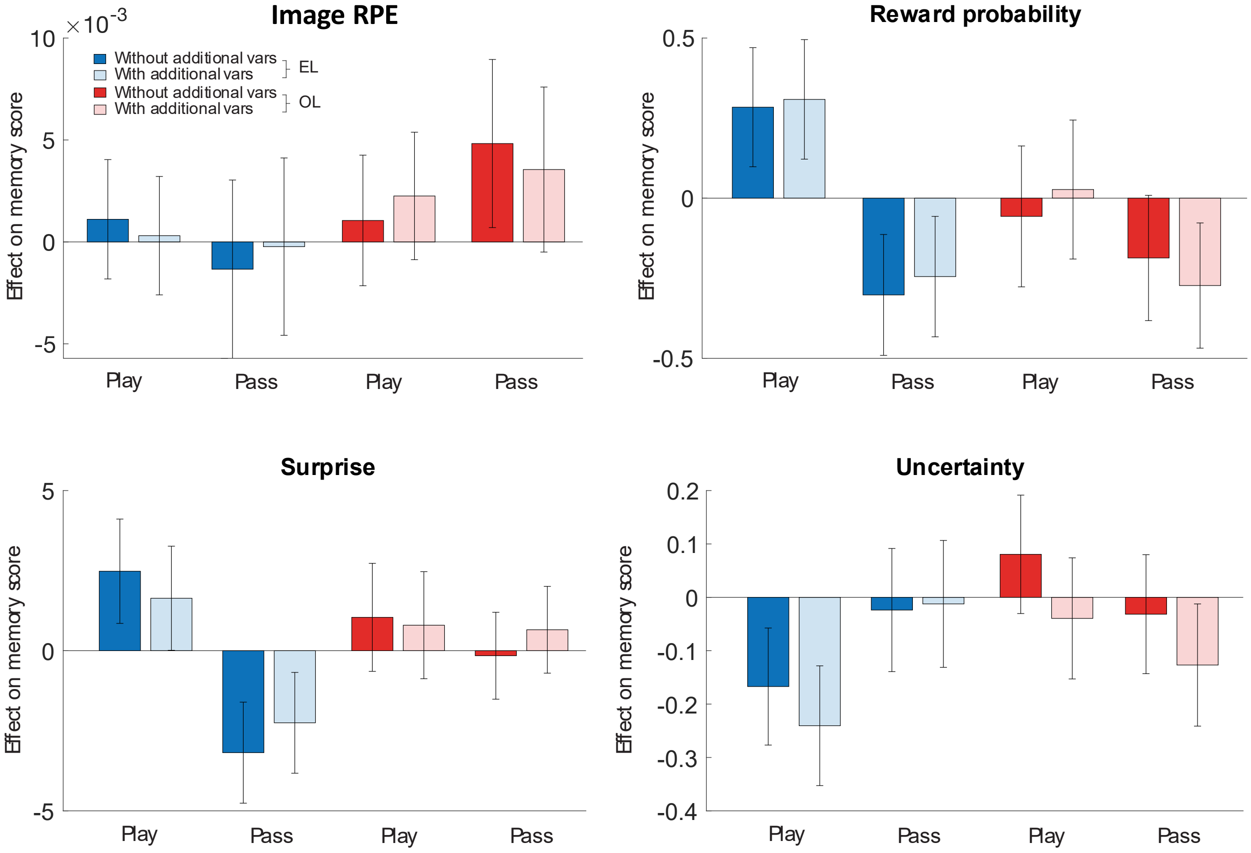

Supplementary Figure 8. Effect of image RPE, reward probability, surprise, and uncertainty on memory score with and without the addition of categorical variables accounting for participant- and image-specific effects. Bars and error bars indicate the regression coefficients and their standard errors, respectively. Blue indicates EL trials, while red indicates OL trials. Lighter shade of each color indicates the regression results after including the additional categorical variables.

Learning task structure

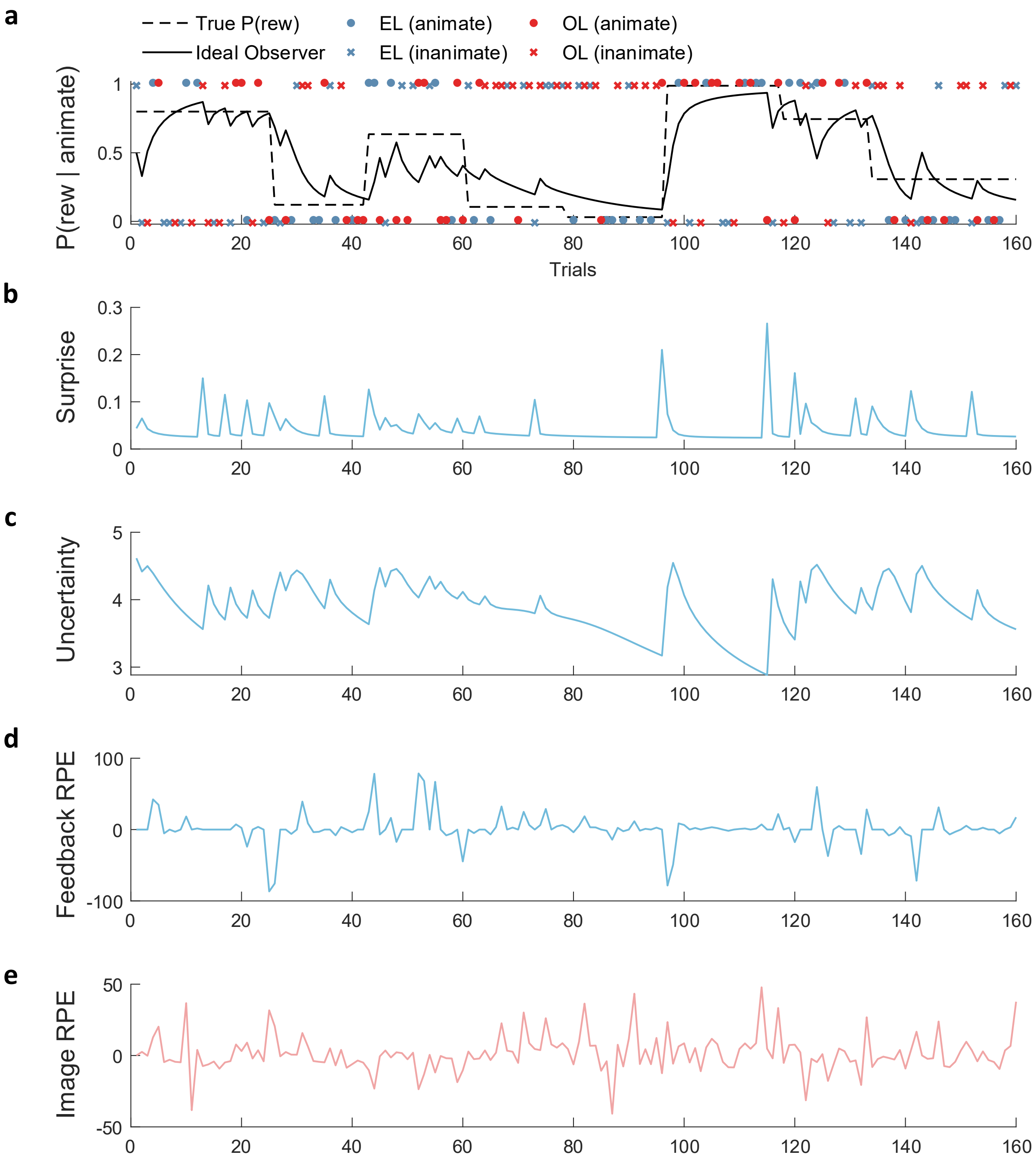

Supplementary Figure 9. Example learning task structure. **a.** Model predictions. Reward probabilities were determined by the image category, yoked across categories, and occasionally reset to require learning (dashed black line). Binary outcomes (blue dots and x in experiential learning (EL) condition, red dots and x in observational learning (OL) condition) depended on the current reward probability and were used as input to the ideal observer model which estimated the underlying expected reward probabilities (solid black line). **b–e.** Ideal observer model. The ideal observer learned from the binary outcomes (1 – reward, 0 – no reward) while considering (**b**) the surprise (change point probability) associated with each trial outcome and (**c**) the uncertainty about the current reward probability. The surprise and uncertainty estimates were dissociable from reward prediction errors (RPEs) elicited during feedback (**d**) and image presentation (**e**).

Other player's gambling behavior

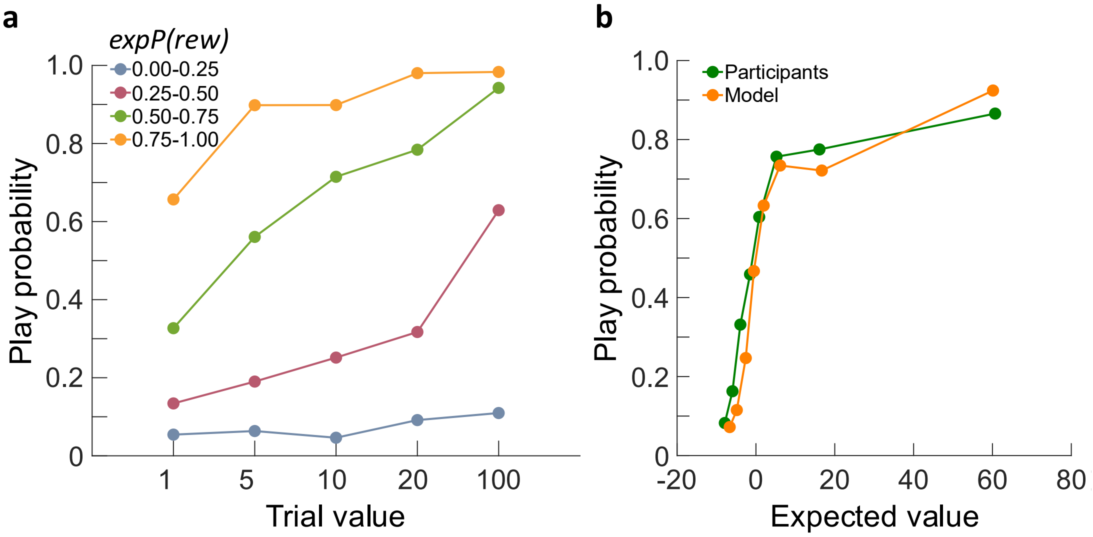

Supplementary Figure 10. **a.** Play probability (n = 79) generated by RL model ("base model") for observational learning (OL) trials (n = 80). Proportion of choosing "play" plotted over trial values [1, 5, 10, 20, 100] and expected reward probability (expP(rew)). **b.** Comparison of participants' gambling behavior and RL model-derived gambling behavior. The proportion of choosing "play" plotted over the expected value. The participants' probability of playing (green) in experiential learning (EL) trials vs. RL model-derived probability of playing (orange) for the same trials (EL).

Memory accuracy

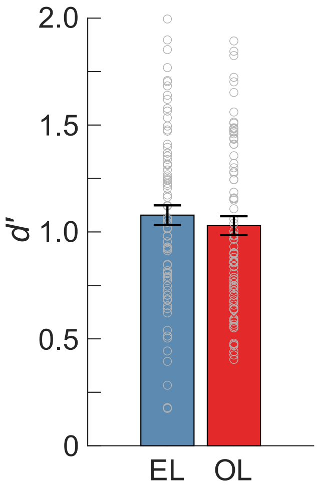

Supplementary Figure 11. Sensitivity index (d', d prime). Average (mean ± SEM) d' values per learning condition (EL, OL) with individual data points (each grey circle represents one participant). Memory accuracy did not differ between learning conditions (paired t-test: t_{78} = 1.19, p = 0.24, d = 0.13, 95% CI = -0.03-0.13).

Proportion of old images vs. memory score

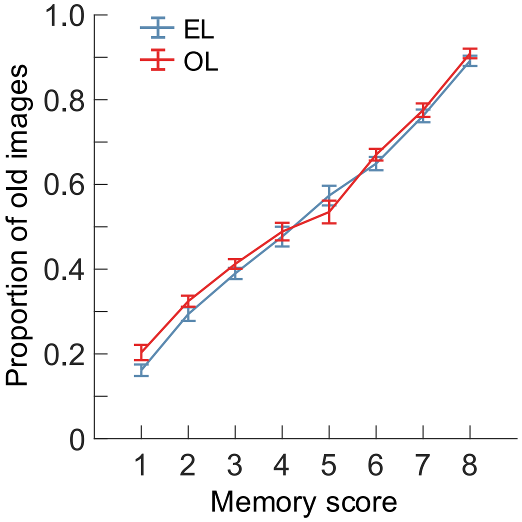

Supplementary Figure 12. Memory score vs. proportion of old images. The true proportion of chosen old images for each memory score (1-8) per learning condition (EL, OL).

Evaluation of participants' and other player's learning task performance

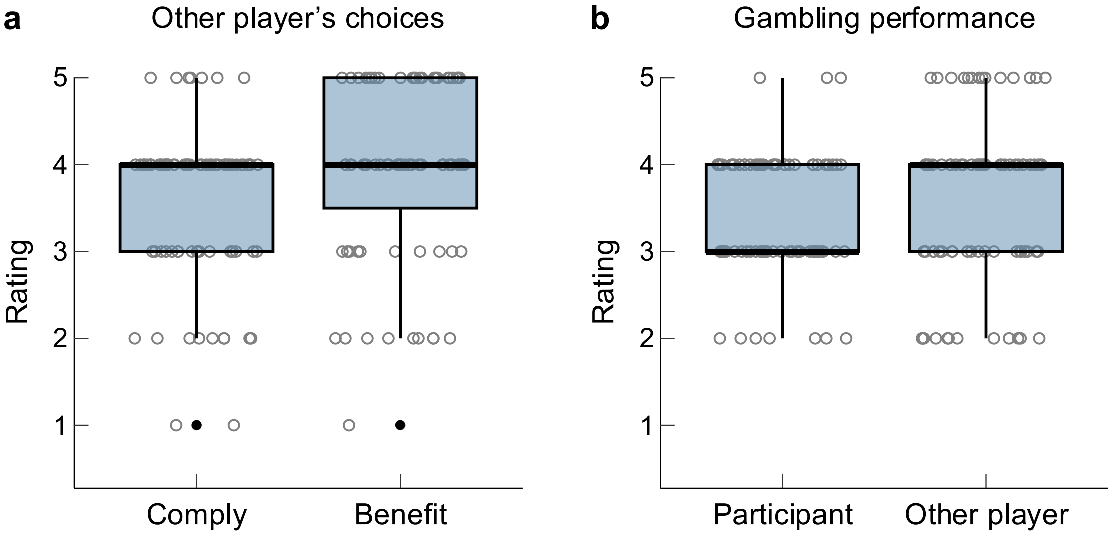

Supplementary Figure 13. Debriefing questionnaire ratings regarding behavior during the learning task. **a.** Participants' ratings (1 – does not apply at all, 5 – applies fully) on whether they followed the other player's choices (OL) and if they benefited from these. **b.** Participants' ratings (1 – very bad, 5 – very good) regarding their own and the other player's performance during the learning task.

Continuous image RPEs

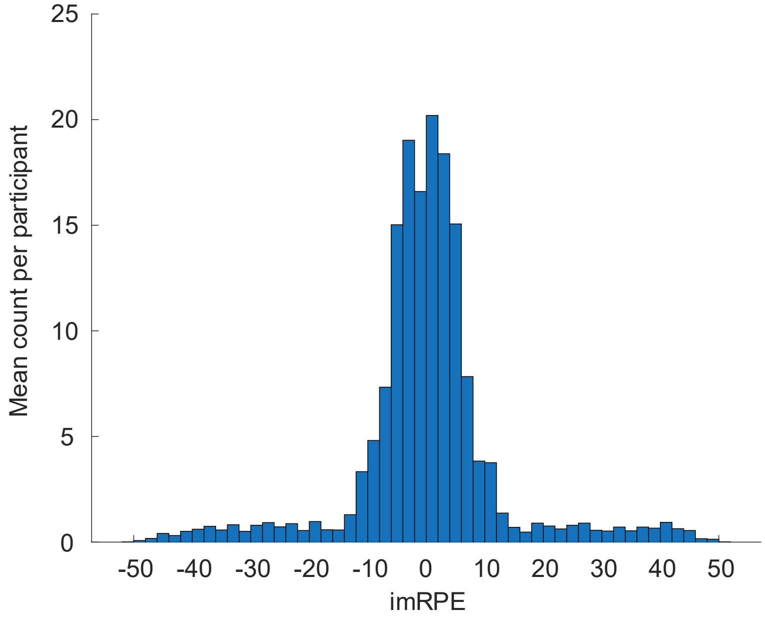

Supplementary Figure 14. Mean trial count per participant, binned by image RPE.

Supplementary Table 21: One-sample t-tests against 0 on the slopes derived from linear regressions fitted to memory scores (old) and continuous image RPEs per learning condition (EL, OL) and choice (play, pass). Corrected α = 0.0125

Memory ~ image RPE	***t***	***df***	***95% CI***	***d***	***p***
EL play	0.22	78	-0.006 – 0.007	0.03	0.82
EL pass	-0.58	78	-0.02 – 0.01	-0.07	0.56
OL play	0.23	78	-0.006 – 0.007	0.03	0.82
OL pass	1.04	78	-0.004 – 0.01	0.12	0.30

Continuous feedback RPEs

Supplementary Table 22: One-sample t-tests against 0 on the slopes derived from linear regressions fitted to memory scores (old) and signed feedback PEs (curr – current, prev - previous) per learning condition (EL play, OL play). Corrected α = 0.0125

Memory ~ signed feedback PE	***t***	***df***	***95% CI***	***d***	***p***
EL play curr	1.84	78	-0.0002 – 0.006	0.21	0.07
OL play curr	0.77	78	-0.002 – 0.005	0.09	0.44
EL play prev	1.03	78	-0.003 – 0.008	0.12	0.31
OL play prev	1.57	78	-0.0009 – 0.008	0.18	0.12

Reinforcement learning model for simulation of other player's gambling behavior

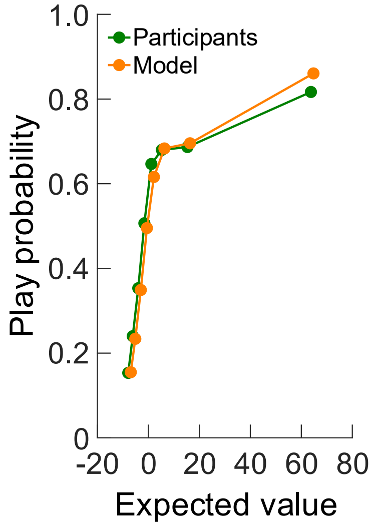

Supplementary Figure 15. Reinforcement learning (RL) model fit. RL model-derived play probability vs. participants' play probability plotted over the expected value. The participants' (n = 30) probability of playing (green) across 80 experiential learning trials vs. play probability (orange) generated by the RL model fitting the participants' choice behavior the best ("base model", lowest BIC) for the same experiential learning trials.

Debriefing questionnaire

1. **How well did you understand the rules of the learning game?**

   Very badly ☐ ☐ ☐ ☐ ☐ very well

2. **How well did you gamble?**

   Very badly ☐ ☐ ☐ ☐ ☐ very well

3. **How well did the other player gamble?**

   Very badly ☐ ☐ ☐ ☐ ☐ very well

4. **Did you comply with the choices of the other player?**

   Does not apply at all ☐ ☐ ☐ ☐ ☐ applies fully

   If yes, when during the game did you do this?

   More towards the beginning ☐

   More towards the middle ☐

   More towards the end ☐

   At all times ☐

5. **Did you benefit from the other player's behavior?**

   Does not apply at all ☐ ☐ ☐ ☐ ☐ applies fully

6. **How did you feel when the other player received a good outcome?**

   Very unhappy ☐ ☐ ☐ ☐ ☐ very happy

7. **How did you feel when the other player received a bad outcome?**

   Very unhappy ☐ ☐ ☐ ☐ ☐ very happy

8. **How well did you remember the images from the learning task?**

   Very badly ☐ ☐ ☐ ☐ ☐ very well

9. **Did you know/guess that there would be a memory test?**

   Yes ☐

   No ☐

References

1. Wilson, R. C. & Collins, A. G. Ten simple rules for the computational modeling of behavioral data. *Elife*, *8*, e49547 (2019).
